# Shotgun Metagenomics Reveals Taxonomic and Functional Shifts in Hot Water Microbiome Due to Temperature Setting and Stagnation

**DOI:** 10.3389/fmicb.2018.02695

**Published:** 2018-11-13

**Authors:** Dongjuan Dai, William J. Rhoads, Marc A. Edwards, Amy Pruden

**Affiliations:** Department of Civil and Environmental Engineering, Virginia Polytechnic Institute and State University, Blacksburg, VA, United States

**Keywords:** drinking water, microbiome, metagenome, metal resistance, hydrogen metabolism, opportunistic pathogens, *Legionella*, premise plumbing

## Abstract

Hot water premise plumbing has emerged as a critical nexus of energy, water, and public health. The composition of hot water microbiomes is of special interest given daily human exposure to resident flora, especially opportunistic pathogens (OPs), which rely on complex microbial ecological interactions for their proliferation. Here, we applied shotgun metagenomic sequencing to characterize taxonomic and functional shifts in microbiomes as a function of water heater temperature setting, stagnation in distal pipes, and associated shifts in water chemistry. A cross-section of samples from controlled, replicated, pilot-scale hot water plumbing rigs representing different temperature settings (39, 42, and 51°C), stagnation periods (8 h vs. 7 days), and time-points, were analyzed. Temperature setting exhibited an overarching impact on taxonomic and functional gene composition. Further, distinct taxa were selectively enriched by specific temperature settings (e.g., *Legionella* at 39°C vs. *Deinococcus* at 51°C), while relative abundances of genes encoding corresponding cellular functions were highly consistent with expectations based on the taxa driving these shifts. Stagnation in distal taps diminished taxonomic and functional differences induced by heating the cold influent water to hot water in recirculating line. In distal taps relative to recirculating hot water, reads annotated as being involved in metabolism and growth decreased, while annotations corresponding to stress response (e.g., virulence disease and defense, and specifically antibiotic resistance) increased. Reads corresponding to OPs were readily identified by metagenomic analysis, with *L. pneumophila* reads in particular correlating remarkably well with gene copy numbers measured by quantitative polymerase chain reaction. Positive correlations between *L. pneumophila* reads and those of known protozoan hosts were also identified. Elevated proportions of genes encoding metal resistance and hydrogen metabolism were noted, which was consistent with elevated corrosion-induced metal concentrations and hydrogen generation. This study provided new insights into real-world factors influencing taxonomic and functional compositions of hot water microbiomes. Here metagenomics is demonstrated as an effective tool for screening for potential presence, and even quantities, of pathogens, while also providing diagnostic capabilities for assessing functional responses of microbiomes to various operational conditions. These findings can aid in informing future monitoring and intentional control of hot water microbiomes.

## Introduction

Hot water plumbing has emerged as a critical nexus of energy, water, and public health. Domestic water heating represents the second largest energy demand in United States homes (Keinath and Garimella, 2017) and a prominent health concern due to the proliferation of opportunistic pathogens (OPs). Many OPs, such as *Legionella pneumophila* and *Mycobacteria avium*, tend to be detected at greater frequencies in hot water systems than in cold water. However, “non-OP” microbes represent the majority of the tap water microbiome and could also have yet undefined human health effects (Edberg and Allen, 2004; Dai et al., 2017). As part of a holistic response to understanding and controlling OPs, there is growing interest in their interactions with the broader microbial community (Baron et al., 2014; Ji et al., 2017; Proctor et al., 2017; Dai et al., 2018). Complex ecological relationships are important for OP proliferation and pathogenicity (Kilvington and Price, 1990; Lau and Ashbolt, 2009; Taylor et al., 2009; Thomas and Ashbolt, 2011). Little work has been done to understand how operational conditions, such as water heater temperature setting and anode rod corrosion, influence tap water microbes. An improved understanding of factors shaping hot water microbiomes could lead to improved management strategies for water and energy sustainability, water quality, and public health.

OPs and the broader hot water microbiomes can be shaped by an array of practical factors. Foremost amongst these are water heater temperature setting and stagnation of hot water in distal pipes. Temperature setting has previously been observed to be an overarching factor driving changes in OP abundance and microbial community composition (Bedard et al., 2015; Rhoads et al., 2015; Ji et al., 2017). Higher temperature (e.g., >55°C) are generally effective for OP control (Bedard et al., 2015), but warm temperatures (e.g., 32–41°C) can stimulate growth of some OPs, e.g., *L. pneumophila* (Rhoads et al., 2015; Proctor et al., 2017). Taxonomic composition, determined by 16S rRNA gene amplicon sequencing, shifted significantly in one study with each incremental increase (2–3°C) from 32 to 53°C (Proctor et al., 2017). Further, higher temperatures (>45°C) can have an overarching effect on microbial community composition and mask the impact of other factors, including pipe material and organic carbon (Proctor et al., 2017). In typical home plumbing and especially in large buildings, it is impossible to maintain water hot at the water heater set point throughout the plumbing or during water use due to heat loss (Fernández-Seara et al., 2007a,b; Bedard et al., 2015). Stagnant water in distal taps is characterized by greater concentrations of bacteria and OPs (Rhoads et al., 2015; Lu et al., 2017) and a distinct microbial composition relative to recirculating hot water (Ji et al., 2017).

Besides temperature and stagnation, elevated trace metals in hot water are also likely important. Trace metals are a defining feature of local water chemistry interacting with plumbing components. Metals are released into bulk water from pipe corrosion and other sources (Gonzalez et al., 2013), but can be elevated further in hot water systems due to anode corrosion and higher temperatures (Andrei et al., 2003; Brazeau and Edwards, 2013). Metals are also of interest given their association with antimicrobial resistance. A range of effects of metals (e.g., copper, iron, and zinc) on hot water microbes have been reported (Kusnetsov et al., 2001; Borella et al., 2004; Rakic et al., 2012; Rhoads et al., 2017). One well-illustrated example is that copper pipe does not universally exert uniform antimicrobial properties and varies dependent on pipe age, biofilm, temperature, and various water chemistry factors (Lu et al., 2014; Rhoads et al., 2017). While increased metal concentrations can directly affect OPs and the broader microbial community composition (Borella et al., 2004; Brazeau and Edwards, 2013; Lu et al., 2014; Dai et al., 2018), few studies have examined the impact on microbial function in detail. Here, we hypothesize that elevated metals in hot water plumbing will select for metal resistance genotypes in the microbial community.

Hydrogen evolution in hot water systems also has profound implications, but has been virtually unexplored. In particular, conventional electric water heaters are equipped with sacrificial anodes, which preferentially corrode to protect the steel tank from corrosive attack, releasing hydrogen and metals in the process (Andrei et al., 2003; Brazeau and Edwards, 2013; ARICO Plumbing Heating Cooling, 2015). Hydrogen generated during corrosion is a valuable electron donor and energy source for microbial metabolism in nutrient-scarce environments. While a relationship between indicators of corrosion, microbes resistant to metals and/or capable of hydrogen metabolism, and potential indirect effects on OPs has been hypothesized (Rhoads et al., 2017), such relationships remain to be verified.

Major advances have been made in recent years in applying next-generation DNA sequencing toward investigating hot water plumbing microbiomes using an amplicon sequencing (Baron et al., 2015; Ma et al., 2015; Ji et al., 2017; Haig et al., 2018), but characterization of bacterial and archaeral populations may be biased, potentially missing over half of the full diversity (Klappenbach et al., 2001; Hong et al., 2009; Pinto and Raskin, 2012; Sun et al., 2013). Amplicon sequencing also overlooks non-targeted kingdoms (e.g., amoeba, virus) and does not provide direct information about function. Although efforts have been made to predict microbial function from 16S rRNA amplicons (Langille et al., 2013; Ji et al., 2017; Dai et al., 2018), direct examination of functional genes would be ideal. To this end, metagenomic sequencing is gaining ground and has been applied to the study of microbial communities inhabiting an array of environments (Shi et al., 2013; Chao et al., 2015; Mangrola et al., 2015; Pinto et al., 2016). Yet, to our knowledge, metagenomics has not been applied to the characterization of hot water plumbing systems. A keyword search of “hot water” AND “metagenom^∗^” in Web of Science and in PubMed yielded 56 and 62 hits, respectively, many of which were studies of hot springs or hydrothermal vents, while none were true metagenomic studies of hot water premise plumbing (access date June 05 and July 12, 2018).

To gain deep insight into how taxonomic and functional dimensions of hot water microbiomes are shaped by temperature setting, stagnation, and associated shifts in water chemistry, we conducted shotgun metagenomic sequencing of a cross-section of samples collected from a novel pilot-scale hot water plumbing rig. Specifically, identical rigs configured with individual water heaters, recirculating lines, and triplicate distal taps testing varying water use frequencies were constructed of commercially available materials, with one rig (control rig) held at a constant setting of 39°C and the other rig (experimental rig) incrementally increased from 39 to 58°C. A sub-set of samples representing a range in operational conditions and known differences in microbial community response was selected based on prior qPCR- and 16S rRNA gene amplicon sequencing-based examination of these rigs (Rhoads et al., 2015; Ji et al., 2017). In particular, we focused on pathogen gene markers, taxonomic composition, and hierarchical functional profiling, with attention to genes associated with metal resistance and hydrogen metabolism. These findings provide important baseline information about the metagenomic composition of hot water plumbing as it relates to real-world operating conditions.

## Materials and Methods

### Rig Configuration and Sample Collection

Two pilot-scale rigs were built to simulate hot water plumbing systems. A schematic diagram of the rigs is provided in Supplementary Figure S1. Details of rig construction and operation were described previously (Rhoads et al., 2015). Briefly, each rig consisted a household electric water heater, a recirculating line, and replicated distal pipes and taps. Influent water was Town of Blacksburg municipal water passing through granuate activated carbon filters. Triplicate distal taps (water flowing downward) were automatically flushed 1 or 21 times per week. Both rigs were acclimated by running at 39°C (actual temperature of recirculating water) for 5 months. Temperature setting was sequentially elevated to 42, 45, 48, 51, and 58°C, each for 2 to 3 months, in the experimental rig, while the control rig remained constant at a setting of 39°C. Twelve samples were selected for metagenomic sequencing (Table 1), including 2 tap waters at T_0_ (one sample from each rig, both run at 39°C), 1 tap water from experimmental rig at T_1_ (run at 42°C), and 9 samples at T_2_ (influent water, recirculating water, and tap water, experimental rig run at 51°C). Bulk water (500 mL) was sampled from the influent, hot water from the recirculating lines, and water that had stagnated in distal pipes for 8 h (flushed 21 times per week) or 7 days (flushed 1 time per week).

**Table 1:** Background information of samples collected from simulated hot water premise plumbing rig.

Sampling Rig (temperature setting)	Sample Name	Time-point (rig run time until sampling)	Sample location	Stagnation time (flush frequency)
Experimental rig (39°C)	Exp8h.a	T_0_ (5 months)	Distal tap^∗^	8 h (21 times/week)
Experimental rig (42°C)	Exp8h.a	T_1_ (8 months)	Distal tap	8 h (21 times/week)
Experimental rig (51°C)	ExpR	T_2_ (13 months)	Hot water recirculating line	0 stagnation
	Exp8h.a	T_2_ (13 months)	Distal tap	8 h (21 times/week)
	Exp8h.b	T_2_ (13 months)	Distal tap	8 h (21 times/week)
	Exp7d	T_2_ (13 months)	Distal tap	7 days (1 time/week)
Control rig (maintained at 39°C)	Ctr8h.a	T_0_ (5 months)	Distal tap	8 h (21 times/week)
	CtrR	T_2_ (13 months)	Hot water recirculating line	0 stagnation
	Ctr8h.a	T_2_ (13 months)	Distal tap	8 h (21 times/week)
	Ctr8h.b	T_2_ (13 months)	Distal tap	8 h (21 times/week)
	Ctr7d	T_2_ (13 months)	Distal tap	7 days (1 time/week)
Influent water	Influent	T_2_ (13 months)	Municipal drinking water (10°C)	NA


*^∗^Water in distal tap cooled to room temperature (26°C) within 30 min after every flush and stagnated until being sampled; NA, not applicable.*

### DNA Extraction, Metagenomic Sequencing, and qPCR

Water samples were immediately filtered though 0.22 μm membrane filters (cellulose ester, Millipore, Billerica, MA, United States) and stored at -20°C before DNA extraction. DNA was extracted from membrane filters using a FastDNA Spin Kit (MP Biomedicals, Solon, OH, United States) following the manufacture’s protocol. DNA sequencing libraries were prepared using the Nextera XT DNA Library Prep Kit (Illumina) and sequenced with an Illumina Hiseq 2500 using the rapid run mode (2× 100 bp) at the Biocomplexity Institute of Virginia Tech. The numbers of total bacteria and *L. pneumophila* were measured from 10-fold diluted DNA extracts using qPCR. Details of target genes, primer sets, qPCR reactions and running were described previously (Wang et al., 2012).

### Water Chemistry

Water samples from the recirculating line and influent were aliquoted for the measurement of aqueous metal concentrations using inductively coupled plasma-mass spectrometry (ICP-MS) following methods 3030 D and 3125 B (Rice et al., 2012). Briefly, samples were acidified with 2% (v/v) nitric acid for a minimum of 16 h prior to the ICP-MS analysis. Blanks and positive controls were processed every 10 samples for internal quality control.

### Metagenomic Sequence Analysis

Sequences were analyzed with the MG-RAST pipeline (version 3.3.6) (Wilke et al., 2016), where paired ends were joined, annotated, and binned. Sequence reads were annotated to the M5NR and RefSeq databases for taxonomy identification, and to Subsystems database for function identification, with a maximum *e*-value of 1e^-5^, 60% minimum identity, and 15 amino-acid minimum alignment length. Representative hit classification was used to quantify taxonomic abundance. PCoA coordinates, tables of taxa abundance based on annotated genes, and level-1, -2, and -3 hierarchical functions were exported for further analysis and visualization. Level-1 function means a course classification of gene functions into 28 categories [e.g., carbohydrate metabolism, virulence disease and defense (VDD)]. Level-2 functions include a finer classification of gene functions within each level-1 function (e.g., VDD is further classified into adhesion, detection, resistance to antibiotics, etc.). Similarly, level-3 is a further classification within each level-2 category. Shannon index was transformed from the α-diversity given by MG-RAST using the following formula: Log_10_ (α-diversity)/Ln(10). The α-diversity was an abundance-weighted average of annotated species from all annotation source databases used by MG-RAST. Relative abundance of a certain taxa is the count of reads annotated as the taxa normalized by total read counts passing the quality control of MG-RAST. Most abundant genera were selected if their relative abundances >0.05% in at least four of the twelve samples. The proportion of a function is the count of reads annotated as encoding the function normalized by total read counts passing the quality control. Sequence reads annotated as *L. pneumophila* were extracted from MG-RAST. A random subset of these reads was searched against the nr database using blastn with the program optimized for highly similar sequences (megablast) to check the taxonomy of the top 100 to 200 hits (through NCBI web access^1^). Sequences were also annotated to Silva SSU database for taxonomy identification (rRNA reads based annotation) for the purpose of comparing with the method of full protein-based taxonomy annotation, from which taxonomy composition results were generated and presented in this study.

Metal resistance and antibiotic resistance were further analyzed in MetaStorm using the read matching (unassembled) pipelines by annotating to the BacMet (v1.1-4) and CARD (v1.0.6) databases, respectively (Arango-Argoty et al., 2016). The most abundant 50 antibiotic resistance ontologies (AROs) were manually categorized based on target antibiotics and mechanisms of predicted resistance. Metal resistance abundance was grouped by the target metal(s). Relative abundances of interested resistance were normalized to 16S rRNA reads to estimate gene copies per genome or normalized to reads per kilobase of transcript per million mapped reads (RPKM) to counteract differences in protein length and sequencing depth when comparing among samples. The metagenomic data was deposited in MG-RAST^2^ (project SLOAN1) and in European Nucleotide Archive database (accession number PRJEB27971).

### Statistical Analysis

Statistical comparisons of the proportions of hierarchical functions among samples or among groups of samples were conducted in STAMP (Parks et al., 2014). Difference between proportions (DP) was used to conduct statistical tests. Briefly, ANOVA with Benjamini–Hochberg FDR correction methods for multiple tests was used to compare among multiple groups, followed by Tukey–Kramer *post hoc* tests. Welch’s *t*-test with Storey’s FDR or Benjamini–Hochberg FDR correction for multiple tests was used to compare two groups of samples, using DP: Welch’s inverted method to calculate 95% confidence intervals. Fisher’s exact test with Storey’s FDR or Benjamini–Hochberg FDR correction for multiple tests was used to compare two individual samples, using DP: Newcombe-Wilson to calculate 95% confidence intervals. A *p*-value < 0.05 and RP > 1.05 (relative difference >5%) were used to select functions to be displayed.

## Results

The cross section of 12 samples representing key hot water plumbing operational conditions (Table 1) were divided between two Illumina lanes. Each sample achieved 1.8 to 8.9 million stitched reads that passed MG-RAST quality control. The average read length was consistant across samples (mean = 143 to 149 bp). Estimated sequencing coverage varied from 80 to 91% according to the Nonparel curves from Rodriguez-R and Konstantinidis (2014), meeting the two criteria (>60% coverage, < 2-fold difference in coverage among samples) for comparative metagenomic analysis as suggested by their study (Rodriguez-R and Konstantinidis, 2014). The proportion of reads successfully annotated varied from 63 to 80% among the samples. Recirculating hot waters yielded significantly more annotated reads (79 ± 2%, *n* = 2, *p* = 0.03) than distal tap waters (69 ± 3%, *n* = 9) or the influent cold water (63%). Taxonomy was assessed based on all annotated functions, rather than solely rRNA genes, which composed less than 0.5% of all annotated reads in each sample. All samples had highly similar distributions among kingdoms, where Bacteria dominated (98.7 ± 0.3%) with a minority composition of Archaea (0.3 ± 0.1%), Eukaryota (0.9 ± 0.3%), and Viruses (0.06 ± 0.02%). The dominance of Bacteria in our hot and cold water samples is consistent with what has been reported from previous metagenomic studies of drinking water (Shi et al., 2013; Chao et al., 2015), while Archaea can have a larger (>5%) proportion in hot spring samples (Chan et al., 2015; Mangrola et al., 2015).

### Overall Impact on Microbial Composition and Predicted Function

**FIGURE 1:**
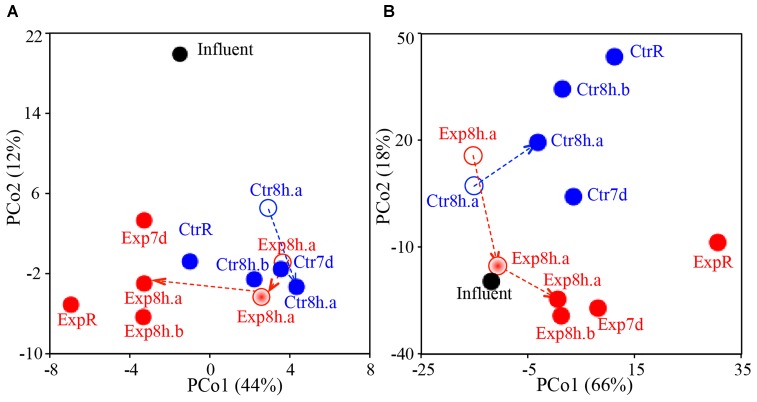
PCoA plots comparing overall similarities in **(A)** taxonomic structure and **(B)** functional gene composition as a function of time, temperature, and stagnation. Blue, red, and black data points represent samples from the control rig (Ctr), experimental rig (Exp), and influent cold water, respectively. Open, half-solid, and solid circles represent samples from T_0_ (both rigs run at 39°C), T_1_ (experimental rig run at 42°C), and T_2_ (experimental rig run at 51°C), respectively. Individual data points are labeled with “R” to indicate samples from the recirculating line, with stagnation time (8 h, 7 d) to indicate samples from distal taps, and with letters (a, b) indicating tap replicates. Dashed arrows correspond to the time series as samples were collected from the same tap from T_0_, T_1_ to T_2_.

Overall similarities across the samples in terms of their taxonomic and predicted functional compositions are illustrated via PCoA plots (Figure 1). Initial microbiome profiles of distal tap water in the experimental and control rigs were markedly similar at T_0_ (i.e., samples Ctr8h.a and Exp8h.a at T_0_ are located in closer proximity to each other in Figure 1, Bray–Curtis similarity was 95–98% between these two samples vs. 79–94% between other sample pairs). This indicates that the rigs had reached a similar baseline after 5 months of acclimation at 39°C. The two sets of replicated distal taps (a vs. b at T_2_ in both rigs) were also highly similar (similarity = 96–99%). These highly similar baselines and replicates facilitated subsequent comparisons between the two rigs and between individually sequenced samples for characterization of temperature and stagnation impact. Notably, temperature setting and stagnation induced stronger shifts (shown as longer distances between ExpR and CtrR or between recirculating and stagnated waters on PCoA plots) in the microbiome profiles than the actual period of stagnation (shown as shorter distances between stagnated waters for 8 h or 7 days, Figure 1).

Water heater temperature setting not only shaped the microbiome inhabiting the recirculating hot water line, which maintained the set temperature, but also distal tap waters after they had cooled during stagnation to room temperature. Incrementally elevated temperature setting in the experimental rig resulted in greater dissimilarities in tap water (T_0_ to T_2_, dissimilarity = 12%) than what could be accounted for based soley on temporal effects (T_0_ to T_2_ in control rig, dissimilarity = 7%). A prior study of these rigs indicated that the hot water entering these pipes cooled to room temperature within 30 min (Rhoads et al., 2015). This cooling and stagnation resulted in a distinct taxonomic and functional composition relative to the recirculating line at both temperatures, with functional shifts especially prominent at higher temperature setting (dissimilarity = 12–15% at 51°C vs. 7–11% at 39°C). Extended stagnation from 8 h to 7 days showed the least impact on microbial composition and function (dissimilarity = 5–7%, Figure 1). Also striking was the distinct clustering of influent cold water away from all hot water samples in taxonomy composition (Figure 1A), indicating strong taxonomic shifts induced by water heating. However, similar distinct clustering was not observed in annotated functions, where influent water was similar to distal tap waters on Figure 1B. Similar functional compositions among distinct taxonomic compositions may result from functional redundancy, especially at higher hierarchy levels, among different taxa. Adonis analysis (Supplementary Table S1) confirmed that temperature setting exhibited the greatest impact on the taxonomy and function of hot water microbiomes (*R*^2^ = 0.411 and 0.245, respectively), followed by stagnation (recirculating or stagnated, *R*^2^ = 0.136 and 0.052) and the period of stagnation (stagnated for 8 h or 7 days, *R*^2^ = 0.067 and 0.021).

### Impact of Temperature Setting

Impact of temperature setting was explored in recirculating hot water samples that remained at the set point. Distinct taxa were selectively enriched at different temperature settings (39°C vs. 51°C), while changes in the proportions of genes encoding various cellular functions were apparent at the top hierarchy level.

The relative abundance of the thermophilic phylum, namely Deinococcus–Thermus, increased when influent cold water (relative abundance 4%) was heated in water heaters more sharply at higher temperature setting (22% at 39°C, 30% at 51°C, Supplementary Figure S2A). Meanwhile, Proteobacteria decreased in hot waters more markedly at higher temperature setting (from 73% in influent to 62% at 39°C and to 50% at 51°C, Supplementary Figure S2A). Sub-orders of Proteobacteria exhibited different responses to temperature setting (e.g., *Burkholderiales* β-Proteobacteria has lower relative abundance at higher setting, while other β-Proteobacteria exhibited an opposite trend, Supplementary Figure S2B), suggesting that analysis at finer resolution (such as at genus level) was needed to reveal compositional shifts associated with temperature.

**FIGURE 2:**
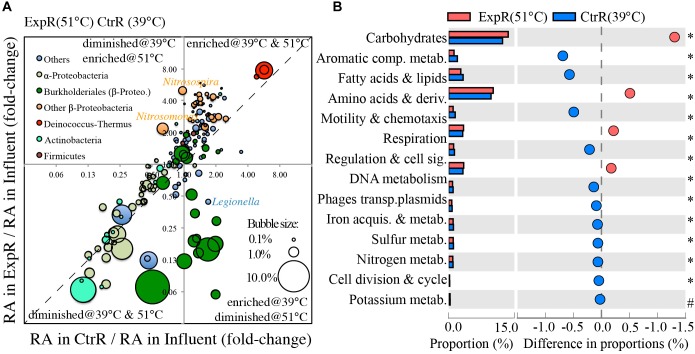
Effect of temperature setting on genera and functions of the microbiota inhabiting hot water recirculation lines. Temperature setting (39°C vs. 51°C) affects **(A)** fold-change of the relative abundances (RA) of 241 most abundant genera in recirculating hot waters (39°C or 51°C) in comparison to influent water and **(B)** proportions of level-1 hierarchical functions of hot water microbes in recirculating lines. Relative abundance (RA) is the count of reads annotated as the taxa normalized by total read counts. Bubble size in panel **A** reflects the relative abundance of each genus in the influent. In panel **B**: blue and red circles represent functions with a higher proportion in the control (CtrR) and experimental (ExpR) recirculating hot waters, respectively, relative to the other. Proportion is the count of reads annotated as encoding a function normalized by total read counts. The symbol ‘^∗^’ indicates *p*-value < 1e^-15^ and ‘^#^’ indicates *p*-value = 2.28e^-10^ when statistically comparing the two samples using STAMP. Refer to Supplementary Figure S5 for full definition of level-1 functions.

The most abundant genera were selected for further comparative analysis (241 total). The diagonal line in Figure 2A, in proximity to which most genera fell, is indicative of similar responses to water being heated to different temperatures (39 and 51°C). This analysis also highlights a few genera that exhibited much sharper shifts in relative abundances, in response to temperature setting (Figure 2A). Specifically, genera deviating further from the diagonal line responded most dissimilarly to temperature settings. Among them, several *Burkholderiale* genera and a few non-α/β-Proteobacteria genera were inhibited at higher temperature (51°C), but were enriched or less inhibited at a lower setting (39°C), both relative to the influent. The *Burkholderiale* genera were the most abundant ones in cold influent water. Their higher degree of inhibition at 51°C than at 39°C probably resulted in a more even distribution at the genus level, and thus a higher diversity index at 51°C than at 39°C, as shown in Supplementary Figure S3. Also notable among the genera trending away from the diagonal line is *Legionella*, whose relative abundance decreased at 51°C (0.25% in influent to 0.11% in hot water, fold-change of hot-water/influent = 0.44) but increased at 39°C (0.42% in hot water, fold-change = 1.69), agreeing well with a mesophilic phenotype of *Legionella*. In contrast, several non-Burkholderiale genera (e.g., *Methylobacillus, Methylotenera*, and *Methylovorus*) and three Deinococcus–Thermus genera were more enriched at the higher setting (e.g., fold-change = 2.65–4.56 at 51°C vs. 1.14–1.64 at 39°C for methylotrophs, Figure 2), while *Nitrosomonas* and *Nitrosospira* were also enriched at 51°C (fold-change = 2.18–4.98) but inhibited at 39°C (fold-change = 0.63–0.97).

Accompanied with the distinct changes in microbial taxa were changes in the proportion of genes encoding various functions, including those characterizing the top hierarchy (level-1, Figure 2B). More genes were involved in the metabolism of aromatic compounds, fatty acids, and lipids/isoprenoids, as well as in cell motility/chemotaxis when temperature was set lower (39°C). In contrast, genes involved in cellular functions for carbohydrate metabolism, amino acids and derivatives, and respiration had higher proportions at the higher temperature setting (51°C).

Similar impact of temperature setting on taxonomic and functional composition was still observable in distal tap waters, yet less prominently than when comparing recirculating waters (Supplementary Figures S4, S5). Remarkably, the overall microbial diversity (i.e., Shannon Index) was not distinguishable between the two temperature settings in tap waters after 7-day stagnation (Supplementary Figure S3).

### Impact of Stagnation in Distal Pipes

Stagnation of hot water in distal taps (i.e., cooling down + stagnation) diminished taxonomic and functional changes in the hot water microbiome introduced by water heating. For example, microbial diversity decreased when water was heated (Shannon index = 4.9–5.1 in recirculating water vs. 5.5 in influent water), but rebounded after stagnation to reach a similar level as in influent water (Shannon index = 5.3 to 5.6 in tap waters, Supplementary Figure S3). No correlation was found between sequencing depth and diversity estimates (Spearman’s correlation *p*-value = 0.53, Supplementary Figure S6), indicating that varying sequencing depth does not explain the observed differences in diversity. The diminishing impact of stagnation was also observed at the genus level. Stagnation-induced fold-changes in the relative abundance of the 241 genera were negatively correlated with fold-changes caused by water heating, regardless of temperature setting or stagnation period (*p* < 0.0001, Spearman ρ = -0.68 to -0.90, Supplementary Figure S7). This indicates that a genus enriched by water heating would then exhibit a reduction in its relative abundance when hot water stagnates, and conversely, one inhibited by water heating would be enriched after stagnation. Similarly, changes in the proportions of functional categories rebounded following stagnation. Functions with an increased/decreased proportion due to water heating all reversed after stagnation, exhibiting near mirror-like reflective symmetry in Supplementary Figure S8. The diminishing impact of stagnation likely resulted in higher similarities among tap waters from different rigs and time-points. Tap waters were more closely clustered together than with recirculating hot waters (Supplementary Figure S9). Since the diminishing impact was independent of temperature setting and stagnation period, we combined six tap waters from both rigs at T_2_ and compared them with the two recirculating waters for further examination of the impact of stagnation on microbial functions.

**FIGURE 3:**
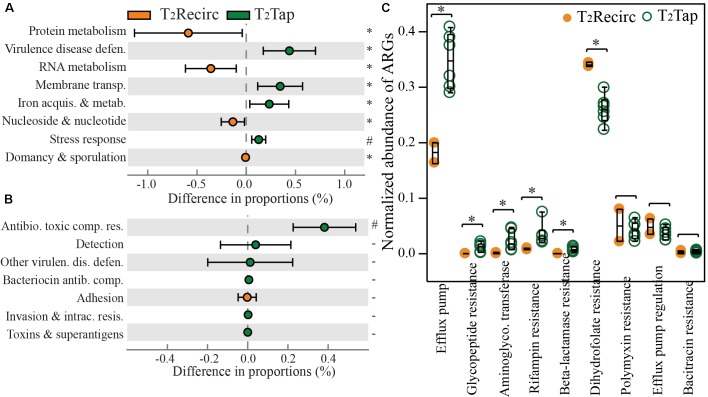
Functional gene shifts following stagnation of hot water in distal taps sampled at T_2_. Comparing recirculating hot waters with tap waters in the proportions of **(A)** level-1 course categories; **(B)** level-2 finer classifications of the virulence disease defense function in panel **A**; and normalized abundance of **(C)** ARGs. T_2_Recirc includes two recirculating waters and T_2_Tap has six distal tap samples, from both rigs at T_2_. Orange and green circles in panel **A** represent functions with a higher proportion in T_2_Recirc and T_2_Tap, respectively. Error bars in panel **A** represent standard deviations. The symbol ‘^∗^’ indicates *p*-value < 0.05 (0.011 to 0.044), ‘^#^’ indicates *p*-value < 0.001, and – indicates *p*-values >0.05 (0.241–0.977) from STAMP analysis. Normalized abundance of ARGs was dividing the count of reads annotated as an ARG by the count of 16S rRNA genes.

Stagnation resulted in a significant reduction in the proportions of genes involved in metabolism and growth, but a significant increase in genes involved in stress-associated cellular functions (*p*-values from < 0.001 to 0.044). Gene proportions for metabolisms (RNA and protein) and growth (nucleosides and nucleotides assembly) were significantly higher in recirculating hot waters than in distal tap waters (DP = 0.38–0.61%, *p* = 0.018–0.044, Figure 3A). In contrast, four functions had significantly higher proportions in tap waters, specifically: VDD (DP = 0.48%), membrane transport (DP = 0.40%), iron acquisition and metabolism (DP = 0.25%), and stress response (DP = 0.10%; *p*-values = 6.87e^-3^–0.033), which are closely related with machineries for cell defense under harsh environments. A major contributor to the largely increased VDD function in tap waters was the only level-2 function: resistance to antibiotics and toxic compounds (Figure 3B). Increased genetic capacity for antibiotic resistance in tap water is deserving of attention as a public health concern. Thus we specifically identified antibiotic resistance genes (ARGs) and normalized ARG counts to 16S rRNA gene counts to assist comparisons among samples. Tap waters contained higher abundances of several ARG categories than recirculating hot waters, regardless of temperature settings (Figure 3C). Two ARG categories, efflux pump and rifampin resistance, increased 1.9 times and 3.9 times, respectively, after hot water stagnates. The near doubling of genes annotated as being involved in efflux pump remarkably agreed with a higher proportion of genes in a related level-1 function: membrane transport (Figure 3A). This is logical, given that many multi-drug efflux pumps were single- or multiple-component membrane transporters (Sun et al., 2014).

### Identification and Quantification of Annotated Pathogen Reads

**FIGURE 4:**
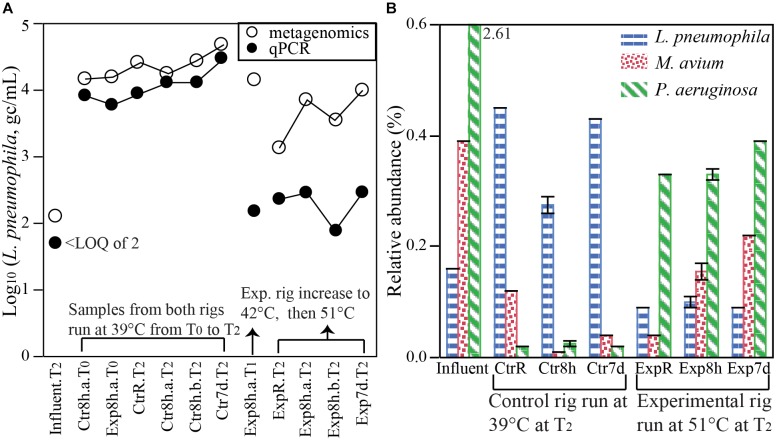
Quantification of OPs DNA sequences in hot water samples. **(A)** Comparing the numbers of *L. pneumophila* in water samples (gc/mL) quantified by metagenomics [relative abundance (%) of *L. pneumophila* (i.e., counts of reads identified as *L. pneumophila /* total read counts) × 16S rRNA gene counts from qPCR quantification (gc/mL)] with *L. pneumophila* numbers from direct quantification by qPCR targeting the *mip* gene. **(B)** Relative abundances of *L. pneumophila, M. avium*, and *P. aeruginosa* (i.e., counts of reads identified as the bacteria species / total read counts in a sample, %) derived from metagenomic analysis. Distinct responses to temperature setting and stagnation are apparent for the three pathogens. LOQ: limit of quantification. Error bars in panel **B** represent standard deviations of two biological replicates.

Pathogens in general and OPs in particular are of special interest among microbes occurring in drinking water. Notably, analysis of metagenomic reads was found to yield remarkably consistent results with the more specific method of qPCR, and even exhibited potential to yield quantitative information. We searched our metagenome sequences for primary and opportunistic waterborne pathogens listed in contaminant candidate list 4 (CCL4) by United States Environmental Protection Agency (EPA, 2016). DNA from nine pathogenic bacterial species were detected (Supplementary Figure S10). Although the cutoff identity was set at 60% by default, the actual sequence identity for OPs such as *L. pneumophila* was found to be much higher (78–95% with a median alignment length of 44 bp) (Supplementary Figure S11). The high identity suggests that this approach to taxonomic classification of these OPs is robust. A blastn search of annotated short reads from MG-RAST (96–183 bp), using *L. pneumophila* as an example, indicated that 75% of reads matched exclusively to this species with >97% coverage of the whole reads and an additional 19% of reads still matching this species at a lower coverage (<50%), further validating the MG-RAST taxonomy assignment pipeline. Besides detects or non-detects, metagenomic analysis also yielded information about the relative abundances of these pathogens of interest. Using *L. pneumophila* as an example, we showed that the relative abundance acquired from metagenomic short reads was potentially reliable for the quantification of absolute numbers as by qPCR (Figure 4A). We calculated the absolute number of *L. pneumophila* by multiplying its relative abundance by total bacterial numbers (measured as 16S rRNA gene copies from qPCR). The numbers of calculated *L. pneumophila* agreed remarkably well with that from direct quantification of *L. pneumophila* gene copies by qPCR in samples that were collected in rigs run at 39°C and in influent (Figure 4A). They also illustrated the same pattern as the qPCR quantifications among different types of samples collected from the experimental rig run with increasing temperature settings (Figure 4A). The results suggest that metagenomic analysis has the potential for the identification and even quantification of target pathogen gene markers at the species level.

Among DNA sequences aligning with the nine bacterial pathogenic species screened in this study, *L. pneumophila, M. avium*, and *Pseudomonas aeruginosa* were the most abundant, while the others were sporadically detected (Supplementary Figure S10). Relative abundances of these three species displayed distinct patterns in responding to temperature setting and stagnation. Specifically, *L. pneumophila* and *P. aeruginosa* changed more dramatically with temperature setting than with stagnation (Figure 4B). The relative abundance of *L. pneumophila* was significantly higher at 39°C in both recirculating hot water and at distal taps, than in influent water or in experimental rigs set at 51°C (Figure 4B). *P. aeruginosa* exhibited the opposite trend, with >10 times more at 51°C compared to 39°C, but it was significantly less in hot waters (0.01 to 0.4%) compared to the influent (2.61%). The response of *M. avium* to temperature setting and stagnation was more complex. Higher temperature setting (51°C) resulted a lower relative abundance in recirculating water but a higher relative abundance after hot water stagnated, indicating an interactive impact between temperature and stagnation. The unique responses of these OPs toward temperature setting and stagnation illustrated the challenge in simultaneously controlling multiple OPs.

Although the samples were prepared for the analysis of bacteria and microbes larger than 0.22 μm, some viral and protozoan pathogenic species on the CCL were also identified (Supplementary Figure S10). Viral DNA may be captured from virus in pre-lysed hosts, lysogenic cycle, or in detached biofilm matrix. *Naegleria fowleri* was rarely (<0.0001%) detected in these samples, suggesting that this warm water-loving brain-eating amoeba is likely absent in the Blacksburg’s cold (10°C) drinking water during the winter season. This is not surprising since *N. fowleri* is more often found in southern-tier states (e.g., Louisiana) during summer months (CDC, 2018). *Acanthamoeba polyphaga* and *Vermamoeba vermiformis* (previously named as *Hartmannella vermiformis*) are frequently reported amoeba hosts of *L. pneumophila. A. polyphaga* was not detected in any sample, but *V. vermiformis* was detected in all samples at low abundances (0.0001–0.0064%). Interestingly, the minivirus genes of *A. polyphaga* were detected in all samples, with a slightly higher relative abundance in hot water samples (0.0003–0.0008%, *n* = 11) than in influent water (0.0002%), suggesting the potential presence of the amoebae host.

Besides *V. vermiformis* and *A. polyphaga*, many other protozoan hosts have been reported to support the multiplication and survival of *L. pneumophila* in water systems (Taylor et al., 2009). Some of these were widely detected in all samples (e.g., *A. castellanii*) and others (e.g., *Dictyostelium fasciculatum*) were sporadically encountered in the metagenomic analysis (Supplementary Figure S12). Positive correlations in relative abundances were found between several of these hosts (e.g., *A. castellanii*, and *V. vermiformis*, *Naegleria* spp.*, Dictyostelium* spp.) and *L. pneumophila* (Spearman ρ = 0.59–0.92, *p* ≤ 0.0001–0.0446; Supplementary Figure S13), suggesting potential commensal relationships.

### Microbial Functions of Specific Interest in Hot Water

**FIGURE 5:**
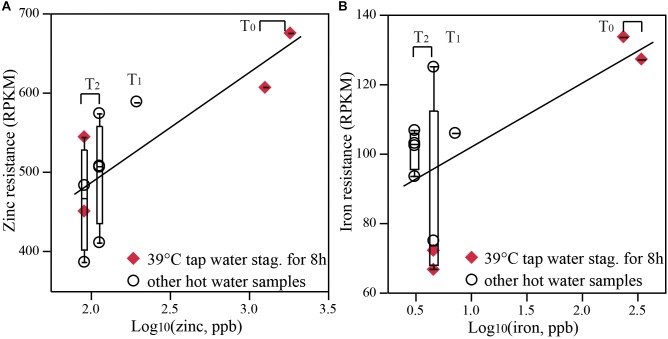
The abundance of metal resistance genes correlated with metal concentration. Normalized abundance of **(A)** zinc and **(B)** iron resistance genes (RPKM, reads per kilobase of transcript per million) in hot water samples correlated positively with zinc and iron levels, respectively, which reduced from T_0_ to T_2_. All distal tap waters and recirculating water from the same rig at the same time point were grouped together for correlation analysis. Correlation coefficient equals to 0.61–0.76, *p* < 0.0001. Distal tap water from both rigs operated at 39°C with a stagnation period of 8 h were labeled as diamonds to emphasize solely temporal change.

With both water chemistry and metagenomic sequence data available, it was possible to test the hypothesis of increased functional genes associated with metal resistance with higher metal concentrations. Genes associated with metal resistance did, in fact, increase, most evidently among hot waters with a temporal shift than among the pair-wise comparisons of hot vs. cold water. The concentrations of various metals (zinc, copper, iron, and lead) increased in recirculating hot waters compared to influent cold water (1 to 42 times higher) at all three time-points for both rigs (Supplementary Table S2). However, leaching of these metals decreased as a function of time. For example, zinc in recirculating hot water in the control system decreased from 1,825.0 ppb at T_0_ (12-times higher than the influent) to 91.6 ppb at T_2_ (only 1.2-times higher than the influent). Concordant with the temporal decrease in metal concentrations was a reduction of genes specifically encoding resistance to zinc and iron (normalized as RPKM in Figure 5 or to 16S rRNA counts in Supplementary Figure S14, correlation coefficient = 0.61–0.76, *p* < 0.0001) and generally toward other metals such as cobalt and cadmium (Supplementary Figure S15) from T_0_ to T_2_. Here we grouped tap water samples with the recirculating hot water from the same time-point together, assuming that distal tap waters had the same metal concentrations as measured from recirculating water. At T_2_, resistance to zinc and copper also trended higher in recirculating water than in influent (Supplementary Figure S16), but statistical confirmation was not possible due to small sample size (2 recirculating waters and 1 influent). Interestingly, corresponding to the only increasing metal from T_0_ to T_2_, magnesium, was the increasing trend of magnesium resistance (Supplementary Figure S14C).

**FIGURE 6:**
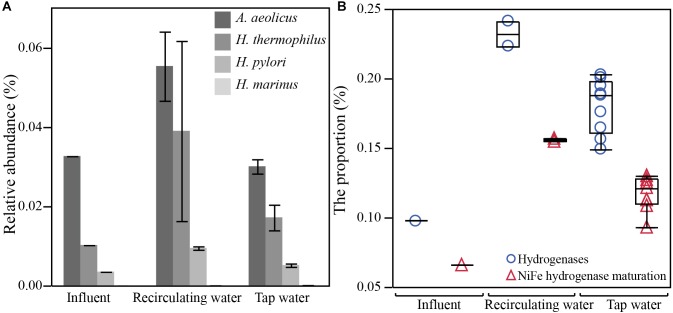
Increased abundance of taxonomic and functional gene markers associated with hydrogen metabolism in hot water. Increased **(A)** relative abundance of four known species of hydrogen-oxidizing bacteria and **(B)** proportion of genes responsible for hydrogen oxidation (hydrogenases and NiFe hydrogenase maturation) in hot waters sampled from the recirculating lines and taps, relative to the cold influent water (Influent). Relative abundance (%) or the proportion (%) was calculated by dividing the count of reads annotated as a particular species or as a gene encoding the particular function by total read counts in a sample. Error bars in panel **A** are standard deviations. The recirculating water group has 2 samples (ExpR and CtrR), and the tap water group has 9 samples from all three time-points. Box-plots in panel **B** show the maximum, 75th percentile, medium, 25th percentile, and minimum values.

Consistent with expectation, we confirmed an increased abundance of genes taxonomically classified as belonging to known hydrogen-oxidizing bacteria (HOB) strains and a higher proportion of genes in hydrogen metabolisms in hot waters compared to the influent. Known HOBs identified from metagenomic sequencing included *Hydrogenobacter thermophilus, Hydrogenovibrio marinus, Helicobacter pylori*, and *Aquifex aeolicus* (Olson and Maier, 2002; Guiral et al., 2012). Three of four species exhibited a higher abundance in recirculating hot waters in comparison to the influent (Figure 6A). Besides these species, other microbes are known to be capable of utilizing hydrogen, including *Escherichia coli*, sulfur oxidizing bacteria, and sulfate reducing bacteria, which have fairly wide taxonomic distributions (Dubini et al., 2002; Anantharaman et al., 2013). Hydrogen-utilizing microbes are expected to contain hydrogenases, a group of key enzymes required for hydrogen metabolism. Two major types of hydrogenase were detected in our samples, Ni-Fe hydrogenase and Fe-Fe hydrogenase with the former one being dominant (77-times more abundant). A third Fe-only hydrogenase was not detected, probably because archaea were rare in our samples and this hydrogenase is only found in some hydrogenotrophic methanogenic archaea (Shima et al., 2008). Notably, the proportion of hydrogenase genes was significantly higher in recirculating hot waters (0.23 ± 0.01%) and in tap waters (0.18 ± 0.03%) than in influent (0.10%) (*p* < 0.05, Figure 6B). Moreover, genes involved in the maturation of the most abundant Ni-Fe hydrogenase were found to be also significantly higher in hot waters (recirculating 0.16 ± 0.001%, taps 0.12 ± 0.01%) than in influent (0.06%, *p* < 0.05). Several genes widely involved in a variety of electron acceptors, such as NAD + /NADH +, cytochrome, quinone, iron-sulfur, sulfur, and coenzyme F420, that are known to pair with hydrogen oxidation were also detected.

## Discussion

This study applied metagenomic sequencing toward characterizing shifts in taxonomic and functional dimensions of premise plumbing hot water microbiomes, with specific attention to water heater temperature setting and stagnation at distal taps. To our knowledge, this is the first application of shotgun sequencing for characterizing hot water microbiomes in premise plumbing, with a particular emphasis on real-world plumbing conditions achieved through controlled, replicated pipe rigs. We found that different water heater temperature settings selectively enriched/diminished distinct taxa and stimulated/inhibited microbes carrying genes corresponding to certain cellular functions. Stagnation in distal taps was associated with diminished taxonomic and functional genes that had initially been selected by consistent, elevated temperatures in the hot water recirculating lines. We demonstrated that unassembled short metagenomic reads were effective for broadly screening for the potential presence, and even quantities, of pathogens of interest. Metagenomic analysis also appeared to be a sensitive indicator of microbial response to various water chemistry factors, supporting hypotheses that elevated metals and hydrogen stimulate selection of corresponding microbes and functional genes. Such information is valuable in moving toward intentional control of building plumbing microbiomes.

Based on prior publications (Rhoads et al., 2015; Ji et al., 2017), a representative cross-section of samples was selected for metagenomic sequencing corresponding to a range of similar and contrasting operating conditions. While replication is rarely reported in metagenomic studies, here we included biological replicates for two sets of samples, as well as the initial time point of the two rigs that had been operated for 5 months at the same initial temperature of 39°C, allowing composite evaluation of baseline variation resulting from replicated operating conditions, DNA extraction, and sequencing. Notably, a high level of consistency was observed among these replicates (1–5% dissimilarity). Comparing samples from the control rig from T_0_ to T_2_ also helped to quantify potential variation introduced by seasonal change in influent water or water chemistry, aging of pipe rig materials (Supplementary Table S2), and maturation of biofilms. All these factors and among-replicates variation in combination (resulting 7% dissimilarity) showed comparable impacts to those introduced by factors of interest in this study (e.g., additional 5% dissimilarity by temperature alone), indicating that differences observed resulted from experimental factors. However, there was insufficient statistical power to confirm many observed differences and thus it is advised to validate any specific patterns of interest observed in this exploratory study with confirmatory experiments.

Taxonomic composition was primarily determined in this study based on phylogenetic identification derived from all protein annotations using unassembled short reads, rather than rRNA sequences, which only accounts for 0.33–0.50% of reads in a sample and among which only 24–33% were annotatable to kingdom level and 10–18% to phylum level. Although both methods have been applied in different studies (Jimenez et al., 2012; Chan et al., 2015; Ghelani et al., 2015), the comparison here suggests that taxonomic annotation based on all available protein features yields improved resolution and is more robust than when based on rRNA reads. Further, annotation based on rRNA reads did not yield detection of Archaea and is not capable of detecting viruses. Among Bacteria, both approaches yielded similar trends only for a few most abundant phyla, but there was zero detection of 11 minor phyla by the rRNA-based approach (Supplementary Figure S17). Another substantial concern is the very low number of rRNA reads available in a typical metagenomic library to profile taxonomic composition. Among millions of reads per sample, only 704–4,058 was used for phylum annotation and <15 for identification of *L. pneumophila*, much less than what is available following most rarefaction subsampling counts applied in amplicon sequencing. Thus we chose to analyze taxonomic composition based on all protein features. Resulting taxonomic composition was found to be similar to those metagenomic-based drinking water surveys or 16S rRNA amplicon studies. For example, we found that Proteobacteria was the dominant phyla in cold water (73.4%), agreeing with a previous metagenomic study of drinking water and several amplicon-sequencing based studies (Lautenschlager et al., 2010; Shi et al., 2013; Pinto et al., 2014; Chao et al., 2015). A decrease in Proteobacteria and increase in thermophilic Deinococcus–Thermus in hot water was also observed previously from amplicon sequencing (Ji et al., 2017; Dai et al., 2018). This agreed well with several previous studies that concluded that metagenomic- and amplicon sequencing-based analyses yield comparable results in terms of major phyla identified, though there may be minor differences in less-abundant phyla (Jimenez et al., 2012; Chan et al., 2015).

We examined impacts of three factors: temperature setting, stagnation, and the period of stagnation on hot water microbiomes. Temperature settings resulted in the greater impact on taxonomic and functional changes in hot water microbiomes. Temperature has been previously reported to have an overarching impact on microbial community structure in amplicon sequencing-based studies. Analyzing a larger set of samples, which included the 12 samples that were the focus of the present study, Ji et al. (2017) similarly concluded that temperature induced a much greater impact on recirculating water (*R*^2^_adonis_ = 0.759) than in distal tap waters (*R*^2^_adonis_ = 0.249). Stagnation and stagnation period (i.e., flush frequency) indicated a much lower magnitude impact (*R*^2^_adonis_ = 0.124 and 0.039, *p* < 0.001). Similarly, temperature also appeared to override the effects of other factors (total organic carbon, pipe material, and temporal change of influent chemistry) on the structure and function of hot water microbial communities characterized by amplicon sequencing (Buse et al., 2017; Proctor et al., 2017). The relative impact of temperature and stagnation on functions of the hot water microbiomes examined here was also generally consistent with functional predictions previously derived from 16S rRNA gene amplicon sequencing data using PICRUSt (Ji et al., 2017), which also indicated that temperature was the most influential factor.

Shotgun metagenomics can provide better resolution, higher sensitivity, and more comprehensive characterization of microbial community structures than 16S rRNA gene amplicon sequencing (Logares et al., 2014; Poretsky et al., 2014). Here, a few genera were identified that responded distinctly to different temperature settings (39°C vs. 51°C), though most genera displayed similar responses to water heating among different recirculating hot waters, relative to comparison with the influent water (Figure 2A). Among those preferentially stimulated at the higher temperature (51°C) included all three *Deinococcus–Thermus* genera and a few non-*Burkholderiales* β-*Proteobacteria* (e.g., *Methylobacillus, Methylotenera, Methylovorus, Nitrosomonas*, and *Nitrosospira*). *Deinococcus–Thermus* are well-known thermophiles. They have been widely detected in hot spring waters and geothermal mats, where temperatures of 60–75°C are not uncommon (Ghosh et al., 2015). Many methylotrophs are known to be thermophilic or at least thermotolerant at temperatures such as 60–65°C (Alawadhi et al., 1989; Semrau et al., 2008). Methylotrophs can use disinfection byproducts and methylated compounds that are widely present in municipal drinking water as their energy and carbon sources (Kronberg et al., 1988; Zamani et al., 2015). Moderately thermophilic *Nitrosomonas* and *Nitrosospira* strains were isolated and confirmed to be ammonia oxidizers, with an optimum growth temperature around 50°C (Golovacheva, 1976; Lebedeva et al., 2005), although most nitrifying bacteria grow optimally at much lower temperatures (25–30°C) (AWWA, 2002). The influent Blacksburg town water is treated with chloramine (Blacksburg, 2018), decay of which can provide reduced nitrogen species to stimulate the growth of nitrifying bacteria. In summary, bacteria selectively enriched at a higher temperature setting (51°C) were likely to be identified as themophilic genera that adapt or survive better at 51°C than at 39°C and also corresponded well with available carbon and energy sources in the water. In contrast, *Legionella* and many *Burkholderiale* genera were preferentially stimulated at the lower temperature (39°C, Figure 2A). The mesophilic genus, *Legionella*, has an optimum growth range from 25 to 40°C (Lesnik et al., 2016). The number of *L. pneumophila*, in particular, displayed a reverse-U curve trend with increasing temperatures from 32 to 53°C, reaching a peak at 41°C, which is consistent with warm temperatures falling within its ideal growth range (Proctor et al., 2017). Even higher temperatures can control or kill *Legionella*, though the specific turnover temperature differed among studies (49°C to 55°C) (Bedard et al., 2015; Rhoads et al., 2015; Ji et al., 2017; Proctor et al., 2017). Similarly, many *Burkholderiales* strains have been noted to be enriched in mesophilic reactors and environments (Kim and Park, 2010; Junior et al., 2017; Subhraveti et al., 2018). The trend identified in this study by metagenomic analysis was similar to a previous study employing semi-batch reactors to simulate water heaters, in which a few different bacterial OTUs were found to have been selectively enriched at each specific temperature based on amplicon sequencing (Proctor et al., 2017).

Stagnation and cooling to room temperature diminished the effects of heating-induced impacts on hot water microbes, regardless of actual temperature setting. As a result, stagnated hot waters were clustered together (Supplementary Figure S9), indicating greater similarities in microbiomes among themselves than with recirculating water. Microbial diversity also rebounded after stagnation, consistent with previous findings based on amplicon sequencing (Ji et al., 2017), and became indistinguishable among various temperature settings after the longest stagnation period (7-days). The cooling of hot water to room temperature was considered to be a critical driver for stagnation-induced community change (Ji et al., 2017). Two other drivers may be changes in water chemistry such as organic carbon and metal concentrations in distal tap hot waters (Salehi et al., 2018), and detached biofilms from distal pipes into stagnated water as biofilms had different community composition and higher diversity than bulk water (Ji et al., 2017). Interestingly, while stagnation had a clear effect on the metagenome, the impact of period of stagnation (8 h or 7 days) was much lower in magnitude. A previous study also noted stagnation time to have the least impact on total microbial numbers in hot water taps among factors investigated (Lipphaus et al., 2014), though it can be critical specifically for the numbers of certain pathogens (Ciesielski et al., 1984; Rhoads et al., 2015). The period of stagnation also impacts cold water systems, where stagnation can induce considerable changes in microbial cell concentrations (HPC, total counts, ATP activity) and microbiome compositions (Ciesielski et al., 1984; Lautenschlager et al., 2010; Ji et al., 2015; Ling et al., 2018). Similar impact of stagnation was also observed in biofilms collected from field-operated premise plumbing systems (Inkinen et al., 2016).

Potential presence of pathogens can be indicated by metagenomic sequences via the identification of genes encoding virulence factors and other protein features unique to a pathogen. It is critical to acknowledge; however, that presence of DNA does not confirm presence of a viable pathogen and culture-confirmation is essential in situations of potential public health concern. Here, pathogen identification was attempted from short reads. This can provide improved specificity relative to classification based on 16S rRNA sequences, as it is often the case that closely related pathogenic and non-pathogenic strains share highly similar 16S rRNA sequences (Poretsky et al., 2014; Srinivasan et al., 2015). We detected DNA corresponding to all nine bacterial pathogens on CCL4 (Supplementary Figure S10). The three most abundant, *L. pneumophila, M. avium*, and *P. aeruginosa*, are among the most often identified OPs from hot water systems (Falkinham et al., 2015). The presence of *L. pneumophila*–specific gene markers in the 12 samples was previously confirmed by qPCR. Interestingly, metagenomic analysis revealed highly similar patterns with respect to how abundance of these OPs shifted in response to factors examined in this study (Figure 4B) and in a manner consistent with prior literature. For example, *L. pneumophila* showed higher relative abundance under warm temperature conditions (39°C) than at the higher temperature (51°C), corresponding to the mesophilic nature of this strain. *M. avium* had a higher relative abundance in distal taps than in recirculating lines and a higher abundance from the experimental rig taps than control rig taps. Similarly, higher numbers in stagnated first draw than in flushed hot water were observed in a bathroom distal tap, with a trend of increasing numbers with temperature (20–49°C) (Dumoulin et al., 1988; Lu et al., 2017). *P. aeruginosa* can grow well at 37–42°C (LaBauve and Wargo, 2012), but less *Pseudomonas* were found in hot water systems characterized by higher numbers of *L. pneumophila* (Borella et al., 2004), similar to what we observed in Figure 4B. While these different OPs do share some common features (Falkinham, 2015), their detections and abundances showed different trends with respect to examined factors (Lu et al., 2017), making it difficult to effectively control all of them using a single uniform strategy. The fact that several positive correlations between *L. pneumophila* and known protozoan hosts were confirmed suggests that the metagenomic approach provides information consistent with known microbial ecological relationships. Overall, the findings were encouraging that shotgun metagenomic sequencing has the potential to serves as a useful, high-resolution screening tool to identify the potential presence of pathogens in water systems.

While shotgun metagenomic sequencing is widely applied to provide estimates of relative abundances (Jimenez et al., 2012; Logares et al., 2014; Chan et al., 2015), here we demonstrated that it is possible to derive absolute quantitative information as well. Using *L. pneumophila* as an example, we showed that metagenomic analysis yielded accuracy in quantifying OP gene markers that was highly comparable to that of direct quantification via qPCR, particularly for the steady-state influent and relatively stable rigs. Although metagenomic analysis seemed to yield higher *L. pneumophila* numbers than qPCR in the experimental rig after temperature elevation (to 42°C and then 51°C), the trend was still highly consistent with that of qPCR when comparing across the samples. One possible reason for the higher estimate could be the incomplete degradation of DNA from dead OPs cells in the rig when higher temperature began to control and kill OPs. The experimental rig was subject to a step-wise increase of temperature setting with time. In contrast to the relatively stable number of *L. pneumophila* gene markers measured in the control rig, the number of *L. pneumophila* decreased with temperature (Rhoads et al., 2015). While DNA from dead cells may persist in the environment (water or biofilm), such DNA is less likely to remain intact (Nielsen et al., 2007; Bylemans et al., 2018). Metagenomic analysis may be more tolerant to DNA modification, snicks, and partial break down than qPCR, because it uses shorter (15 amino acids = 45 bp) virulence genes and proteins with some mismatch tolerance (60% identity) for pathogen recognition, while qPCR relies upon accurate primer binding with a single, intact, and longer (78 bp for *L. pneumophila*) gene fragment. Thus, slightly damaged DNA fragments from dead cells may be picked up by metagenomic analysis but not qPCR, contributing to the observed overestimation of *L. pneumophila* than qPCR in non-steady-state scenarios. Another explanation for the observed discrepancy between metagenomic estimation and qPCR quantification may be an intrinsic limitation of the read and similarity-based metagenomic analysis, which is biased toward model, cultivated, and known microorganisms due to its reliance on reference databases (Simon and Daniel, 2011). *L. pneumophila* is among the well-studied and sequenced bacteria. Thus estimation of its relative abundance in microbial community (many of them are not yet studied or sequenced) tends to be higher than qPCR quantification, as shown for all samples in Figure 3A. Knowledge of thermophilic microbes under elevated temperatures (e.g., >45°C) is expected to be more limited than that of microbes most often cultured in laboratories from 25 to 45°C (Lagier et al., 2015). Thus, there was a tendency toward estimates of greater relative abundance and calculated numbers of *L. pneumophila* after temperature elevation in the experimental rig. Agreement between qPCR and next-generation DNA sequencing (general rRNA amplicon sequencing or genus-specific amplicon) in quantification was reported previously at the sub-order and genus level (Inkinen et al., 2016; Pereira et al., 2017; Dai et al., 2018) and between-method differences were explained as resulting from different primer sets and detection sensitivities.

Elevated metals in hot water can be a health concern in and of themselves, thus ingesting hot water is not advised (EPA, 2015). Here the potential for enrichment of metal resistance genes was also considered. Metal concentrations are known to be elevated in new plumbing systems, where some metals (e.g., copper) may play an antimicrobial role and potentially provide a barrier to OPs proliferation. Here we showed strong evidence of elevated metal resistance genes in earlier samples, which also had elevated metals characteristic of new plumbing systems, with a proportional reduction of metal resistance genes as the system aged and metals decreased (Figure 5). We did not observe a significant difference of some metal resistance genes in hot waters vs. the influent municipal water supply (Supplementary Figure S16), probably due to the limitation of low sample number or great variations among various distal taps. Difference between hot and cold waters may be more readily observed in more stable and well-aged systems, rather than an actively aging system in this study. There is no known direct health concern of elevated metal resistance genes. However, ARGs are frequently associated with metal resistance genes, due to co-selection (Baker-Austin et al., 2006; Seiler and Berendonk, 2012; Di Cesare et al., 2016). Bacterial isolates from drinking water that were tolerant to heavy metals were also found to be resistant to multiple antibiotics (Calomiris et al., 1984). Thus elevated metals may stimulate the growth of antibiotic resistant bacteria in hot water premise plumbing, and could exacerbate potential health concerns associated with pathogens.

This study further provided strong evidence of elevated hydrogen metabolism in hot water microbes. Sacrificial anodes comprised of Al, Mg, and/or Zn alloys are widely used in cathodic protection of exposed steels in water heaters from corrosion (ARICO Plumbing Heating Cooling, 2015). Hydrogen is generated along with sacrificial corrosion of the anode (Andrei et al., 2003; Williams et al., 2013). Hot waters in electric water heaters can contain as high as three orders of magnitude more H_2_ than influent cold water (Brazeau and Edwards, 2013; Dai et al., 2018). Elevated H_2_ was hypothesized to promote the growth of HOB and to stimulate hydrogen metabolism (Morton et al., 2005; Rhoads et al., 2017; Dai et al., 2018). Higher abundance of sequences corresponding to several known HOBs were found in hot waters. Moreover, the elevated proportions of hydrogenase genes as well as of chaperon genes for the correct folding and functioning of hydrogenase provided strong evidence of stimulated hydrogen metabolism in hot water. Identified hydrogenase genes covered different active metallic centers (Fe-Fe, Ni-Fe) and various dislocations (cytochrome, transmembrane, periplasm). This suggested a highly diverse microbial community that can metabolize hydrogen. Microbial consumption of hydrogen was considered to play a critical role in cathodic and anodic metal corrosion processes (Pankhania, 1988), resulting elevated metal concentrations and associated health concerns for ingesting the water and, as shown here, potentially with respect to microbes with high metal resistance and antimicrobial resistance.

Changes of global hierarchy functions matched well with observed compositional shifts of hot water microbiome and changes in microbial physiology. For example, the increased proportion of genes involved in the “metabolism of aromatic compounds” at a lower temperature setting (39°C vs. 51°C, Figure 2B) agreed with a selective enrichment of many genera belonging to the order of *Burkholderiales* in hot water at 39°C (Figure 2A). Many *Burkholderiales* isolates have exceptional capability in biodegrading multiple aromatic compounds and thus they are generally of special interest as drivers of *in situ* bioremediation. Moreover, the impressive catabolic potential of *Burkholderiales* was recently recognized by an analysis of 80 *Burkholderiales* genomes, showing a widespread of genes involved in key pathways to degrade aromatic compounds in this taxa (Perez-Pantoja et al., 2012). Another example is the observed increase in gene functions for “resistance to antibiotics and toxic compounds” and the decrease in protein and RNA metabolisms in stagnated water in comparison to recirculating water. This functional change may be related to different physiology of microbes in water. Stagnated hot water contained more microbial cells than recirculating water, partially due to the detaching of biofilms from pipe surface into the first draw (Lautenschlager et al., 2010). Biofilm cells, even if after breaking down biofilm matrix into single cells, showed increased resistance to many stress factors including antibiotics, toxic chemicals, and disinfection. This may explain an overall increase in proportions of sequences corresponding to stress response and cell defense in stagnated tap water, as observed in Figure 3. Biofilm cells also showed different physiology in comparison to planktonic cells. Many cells switch to a dormant metabolism when grown in biofilms, resulting lower metabolic activities than planktonic cells (Koo et al., 2017). Thus genes for RNA and protein metabolisms that belong to the transcription and translation machineries for active growth and activity were observed to be lower in stagnated water (Figure 3). Limited nutrient availability in stagnated water may also promote a switch to dormancy, while nutrients are replenished in the recirculating line with each inflow of fresh water. In summary, shifts in functional gene composition identified by metagenomics coincided well with the observed taxonomic shifts and speculated physiological shifts.

This study applied metagenomic sequencing to explore the hot water premise plumbing microbiome and its response to different temperature settings and stagnation. Temperature setting was generally found to be a more dominant effect than stagnation or stagnation period. Taxonomic shifts and responses to various conditions corresponded well with observed shifts in functional gene abundances, even at the top hierarchal level. Further, genera selectively enriched at different temperature settings were consistent with established temperature-dependent growth kinetics. Following stagnation of hot water in distal taps, prior effects of water heating began to diminish. Physiological changes in microbes in stagnated water may contribute to the observed higher stress response, including resistance to antibiotics. Metagenomic analysis provided solid evidence for elevated genes associated with metal resistance and hydrogen metabolism in hot waters, especially for earlier sampling dates corresponding to higher aqueous metal concentrations. Here it was demonstrated that metagenomic sequencing can not only provide a global snapshot of taxonomic and functional gene markers and their responses to various shifts in water conditions, it also provides higher resolution information regarding particular pathogens/functions of interest than alternative methods. Unassembled metagenomic reads were successfully used as a screen to identify gene markers for waterborne pathogens. However, it is important to be aware that the read and similarity-based approaches are limited based on available reference sequences and proteins in public databases. While these will improve with time, the approach as applied here was still demonstrated to be more accurate under stable conditions and at lower temperatures. Culture-based techniques would also be critical to follow up on such a screening approach to verify any true public health hazard. Future research may examine potential concerns identified about elevated metal resistance and antimicrobial resistance genes, particularly in newly constructed plumbing systems.

## Data Availability

The metagenomic datasets analyzed for this study were deposited in publicly accessible repository (see section “Metagenomic Sequence Analysis”).

## Author Contributions

DD conducted metagenomic sequencing and data analysis, generated all figures and tables, and drafted the manuscript. WR designed, built, and operated the rigs, collected samples, extracted DNA, provided qPCR data and metal concentrations, and participated in manuscript revision. ME and AP directed the study design, advised in data analysis, and revised the manuscript. All authors approved the final manuscript.

## Conflict of Interest Statement

The authors declare that the research was conducted in the absence of any commercial or financial relationships that could be construed as a potential conflict of interest.

## Footnotes

1 https://blast.ncbi.nlm.nih.gov/Blast.cgi

2 https://www.mg-rast.org

## Abbreviations

ARGs: antibiotic resistance genes
AROs: antibiotic resistance ontologies
CCL: contaminant candidate list
DP: difference between proportions
HOB: hydrogen oxidizing bacteria
ICP-MS: inductively coupled plasma-mass spectrometry
OPs: opportunistic pathogens
qPCR: quantitative polymerase chain reaction
RP: ratio of proportions
RPKM: reads per kilobase of transcript per million mapped reads
VDD: virulence disease and defense
