# Supplementary material for: Shotgun Metagenomics Reveals Taxonomic and Functional Shifts in Hot Water Microbiome Due to Temperature Setting and Stagnation
Source: Front Microbiol. 2018 Nov 13;9:2695. doi: 10.3389/fmicb.2018.02695 (PMC6277882; doi:10.3389/fmicb.2018.02695)
Supplement: Supplementary file 1 [file Data_Sheet_1.pdf]

Shotgun Metagenomics Reveals Taxonomic and Functional Shifts in Hot Water Microbiome due to Temperature Setting and Stagnation

Dongjuan Dai[1], William J. Rhoads[1], Marc A. Edwards[1], Amy Pruden[1]*

[1]Virginia Polytechnic Institute and State University, Via Department of Civil and Environmental Engineering, Blacksburg, VA, USA

Supplemantary tables and figures:

Table S1. Relative impact of three factors on taxonomic and functional dissimilarities among eleven hot water samples

Factors	Hot water microbiome taxonomy		Hot water microbiome function	
	Adonis R^2	p-value	Adonis R^2	p-value
Temperature setting (T)	0.411	0.049*	0.245	0.381
Stagnation (S)	0.136	0.16	0.052	0.682
Stagnation period (SP)	0.067	0.349	0.021	0.869
T:S	0.020	0.747	0.042	0.702
T:SP	0.132	0.147	0.208	0.226

Table S2. Metal concentrations in recirculating and influent water samples

Metals (ppb)	Water	T_0	T_1	T_2	Fold of reduction at T_1 (T_0/T_1)	Fold of reduction at T_2 (T_0/T_2)	Recirc. /Influent at T_2	Exp. /Ctr. at T_2
Zn	Influent	348.1	73.6	74.4				
Zn	Ctr. Recirc.	1,825.0	153.3	91.6	12x	20x	1.2x	
Zn	Exp. Recirc.	1,266.0	195.7	113.9	6x	11x	1.5x	1.2x
Fe	Influent	12.2	0.3	0.1				
Fe	Ctr. Recirc.	237.2	10.9	4.6	22x	52x	41x	
Fe	Exp. Recirc.	344.0	7.2	3.1	48x	110x	28x	0.7x
Cu	Influent	8.0	1.5	1.7				
Cu	Ctr. Recirc.	68.3	30.8	11.0	2x	6x	6x	
Cu	Exp.Recirc.	71.2	10.0	9.7	7x	7x	6x	0.9x
Al	Influent	685.6	35.1	63.5				
Al	Ctr.Recirc.	28,680.0	1,202.5	992.5	24x	29x	16x	
Al	Exp.Recirc.	15,450.0	1,645.3	1,139.5	9x	14x	18x	1.1x
Pb	Influent	0.9	0.1	0.1				
Pb	Ctr. Recirc.	16.0	2.5	0.7	6x	22x	12x	
Pb	Exp. Recirc.	11.5	2.0	0.8	6x	14x	14x	1.1x
Mg	Influent	3,520.0	4,977.6	4,945.3				
Mg	Ctr. Recirc.	3,443.0	4,992.3	4,923.3	0.7x	0.7x	1x	
Mg	Exp. Recirc.	3,484.0	5,091.8	4,894.8	0.7x	0.7x	1x	1.0x

Ctr. Recirc. = Control rig recirculating water sample
Exp. Recirc. = Experimental rig recirculating water sample

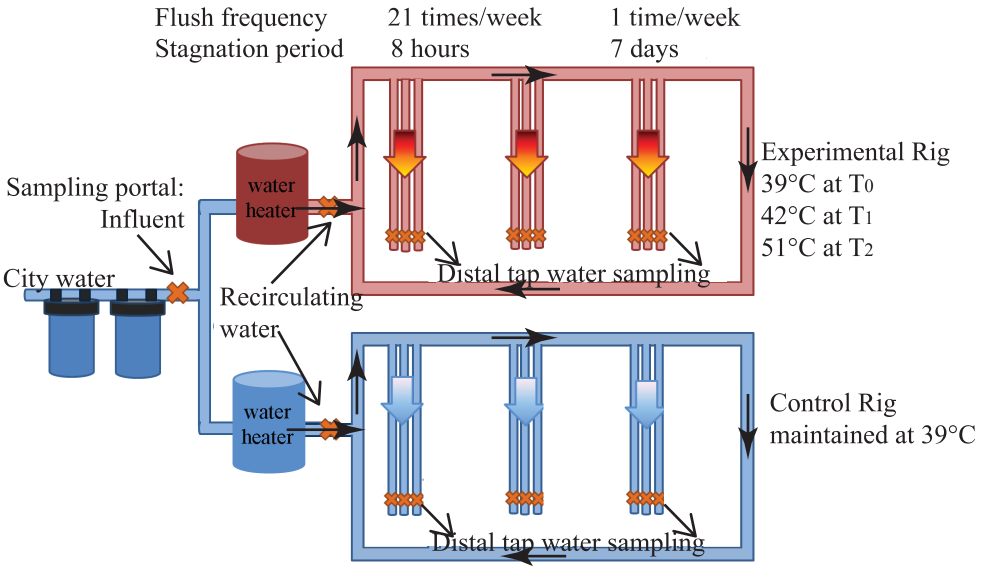

Figure S1. A schematic diagram of simulated hot water premise plumbing rigs. Sampling portals for all samples included in this study, and investigated temperatures in the experimental rigs were labeled.

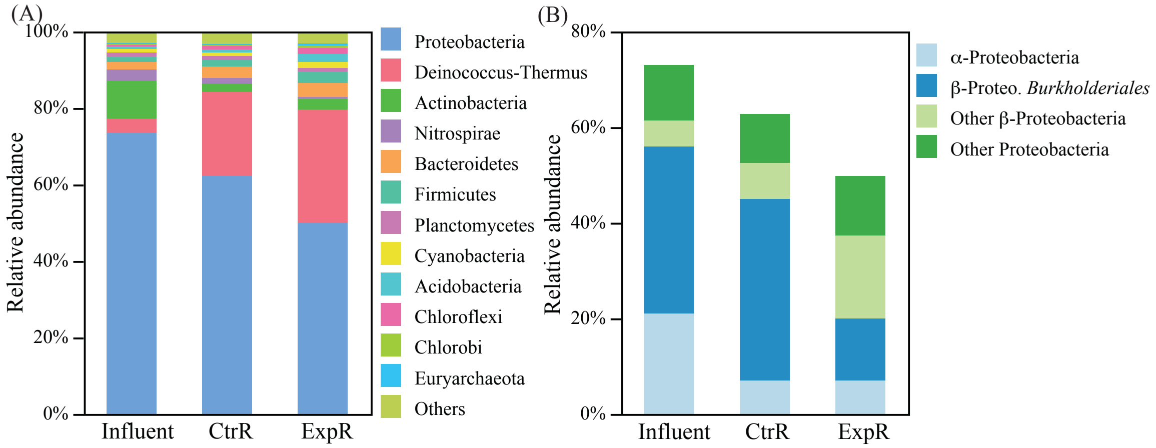

Figure S2. Relative abundance of taxa at the level of **(A)** phyla and **(B)** order within proteobacteria in recirculating hot waters (CtrR: from control rig run at 39°C; ExpR: from experimental rig run at 51°C) and in the influent cold water, all at T_2. Metagenomic reads were annotated to the M5NR and RefSeq databases for taxonomy identification, with a maximum e-value of $1e^{-5}$, 60% minimum identity, and 15 amino-acid minimum alignment length. Relative abundance is the count of reads annotated as one taxa normalized by the sum of all annotable reads.

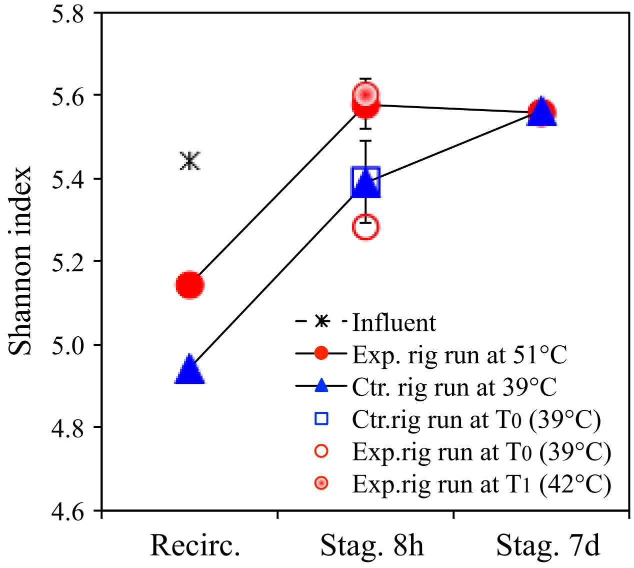

Figure S3. Impact of temperature setting and stagnation on microbial taxonomic diversity (Shannon index). Symbols are color-coded to represent influent water (black asterisk), and samples from experimental rig (red) and control rig (blue). Open, half-solid, and solid symbols indicated time points T_0, T_1, and T_2 respectively. Recirc.: recirculating hot water; Stag.: Stagnation period.

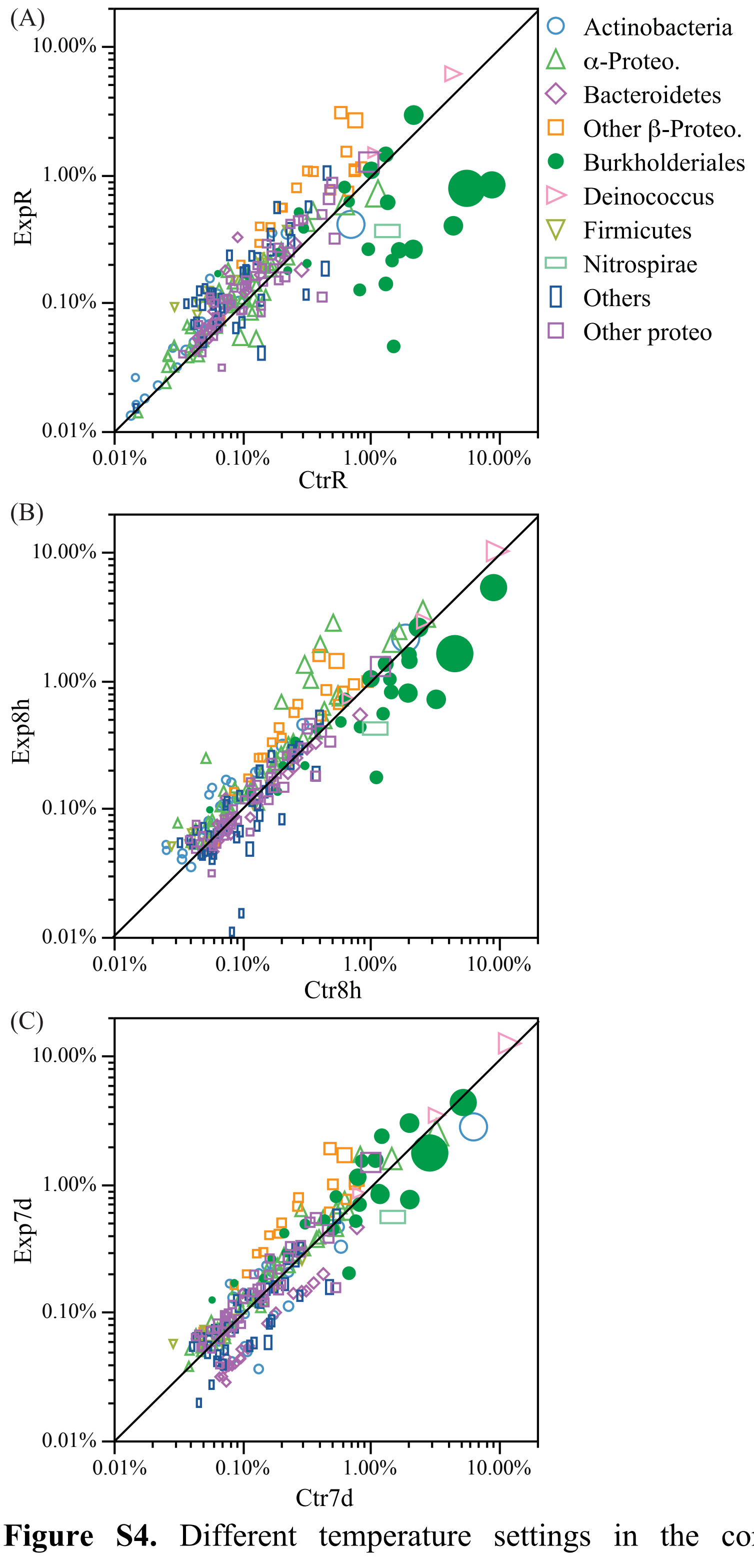

Figure S4. Different temperature settings in the control rig (39°C) and in the experimental rig (51°C) induced differences in relative abundance of 241 most abundant genera in **(A)** recirculating hot water, and tap water stagnated for **(B)** 8 h or **(C)** 7 days in distal taps. Relative abundance of a genus is the count of reads annotated as the genus normalized by total read counts.

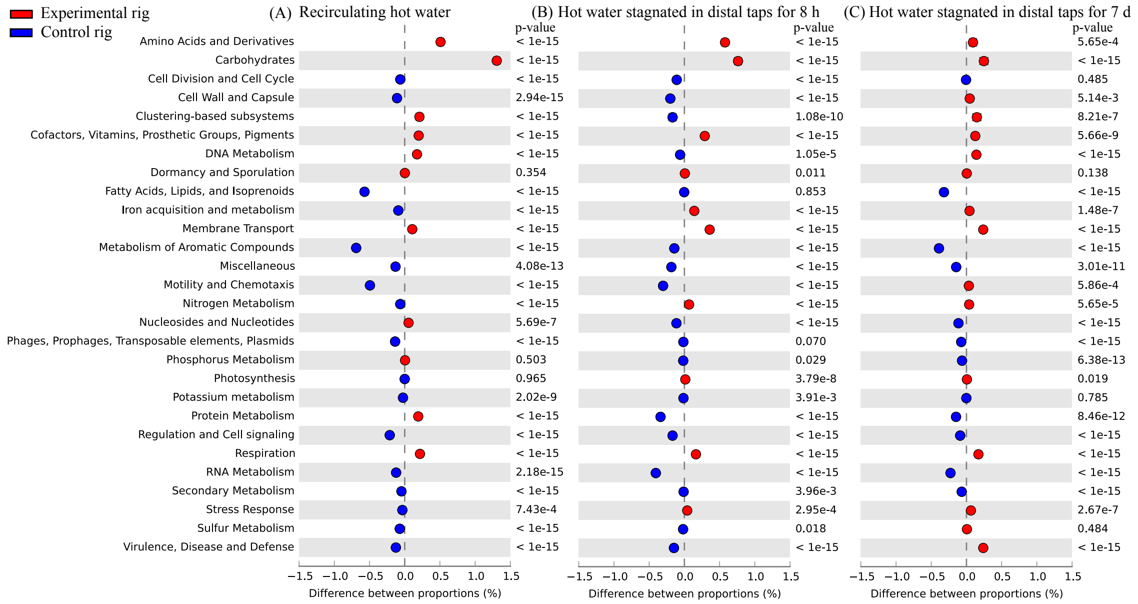

Figure S5. Different temperature settings (39°C in control rig vs. 51°C in experimental rig) induced changes in the proportion of all level-1 functions in **(A)** recirculating hot water, and in hot water stagnated in distal taps for **(B)** 8 h or **(C)** 7 days. The p-values were from two individual sample comparisons. Red and blue circles represent functions with higher proportions in the sample from the experimental rig and control rig, respectively, when comparing the two samples.

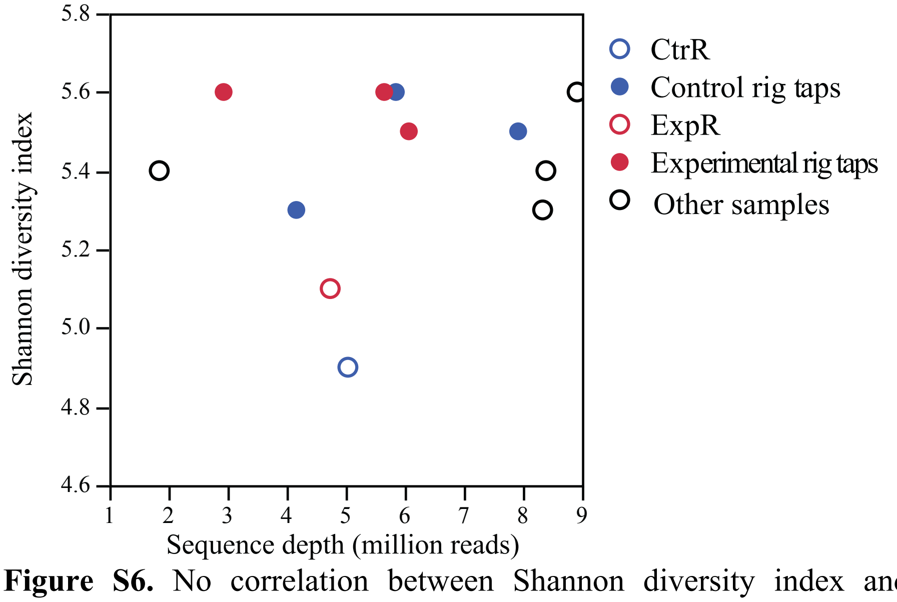

Figure S6. No correlation between Shannon diversity index and sequence depths. Recirculating (open symbols) and distal tap water samples (solid symbols) from the control rig (blue symbols) and experimental rig (red symbols) were highlighted.

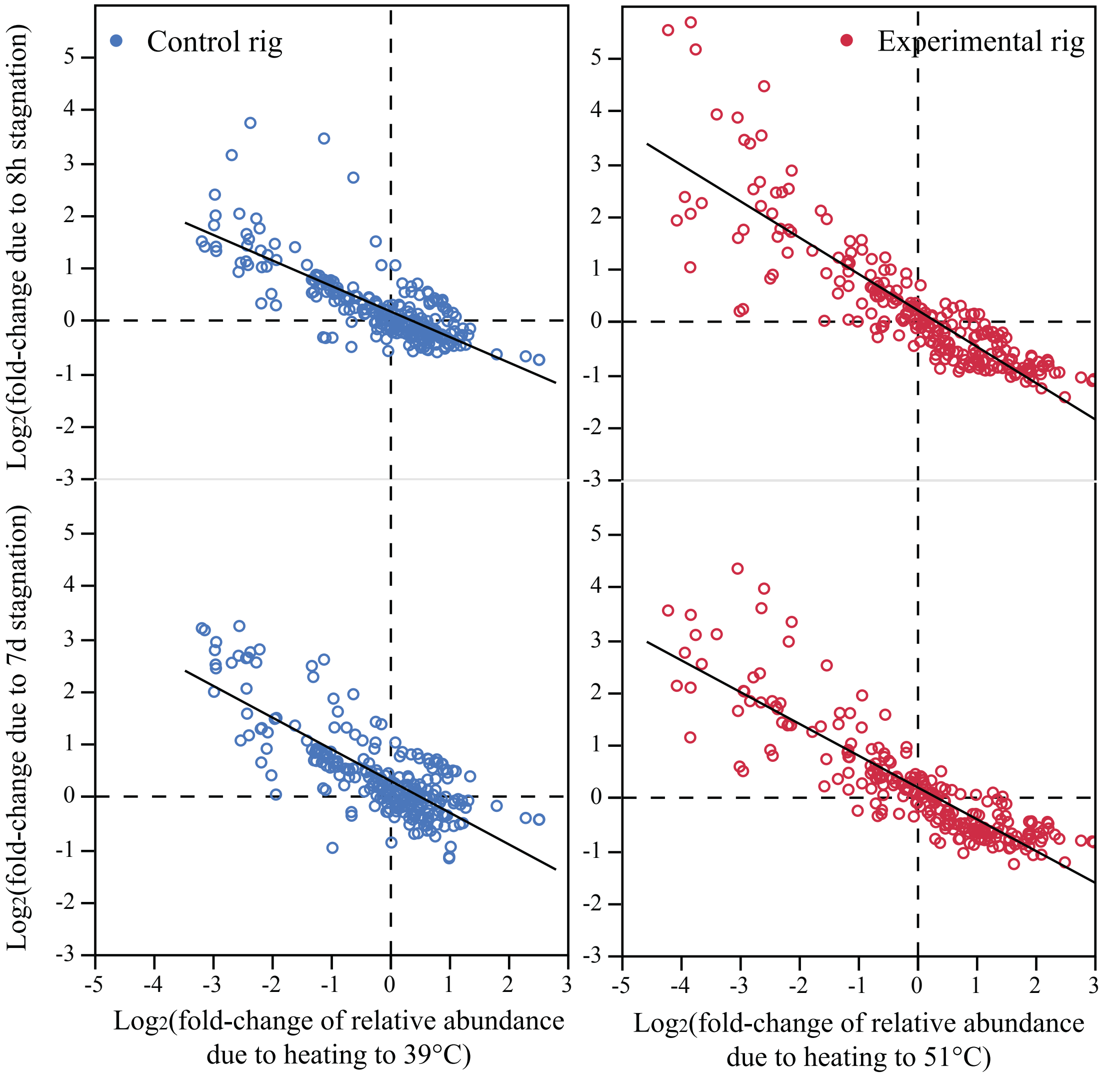

Figure S7. Negative correlations between the fold-change (log-2 transformed) of relative abundance for the 241 most abundant genera due to stagnation (fold-change equals to the relative abundance in tap water divided by the relative abundance in recirculating water) and the fold-change due to water heating to 39°C (blue) or 51°C (red) (relative abundance in recirculating water divided by relative abundance in influent). P-values all < 0.05.

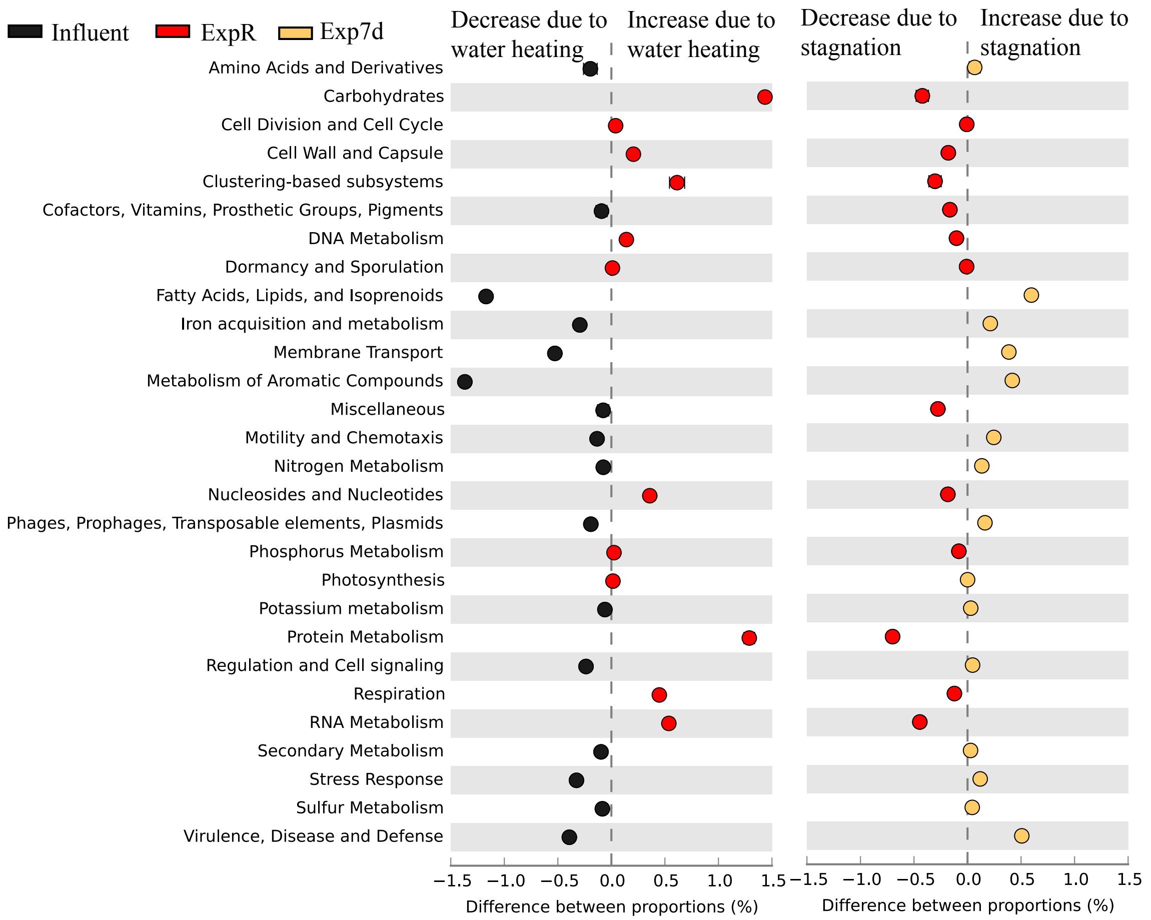

Figure S8. Stagnation overrides water heating-induced changes in the proportions of level-1 functions. Functions with increased proportions due to water heating (red circles in left panel) were also the ones with reduced proportions after stagnation (red circles in the right panel). Red and black circles in left panel represent functions with a higher proportion in the ExpR and Influent sample, respectively, when comparing the two samples. Red and orange circles in the right panel represent functions with a higher proportion in the ExpR and Exp7d sample, respectively, when comparing the two samples. Metagenomic reads were annotated to Subsystems database for function identification, with a maximum e-value of $1e^{-5}$, 60% minimum identity, and 15 amino-acid minimum alignment length.

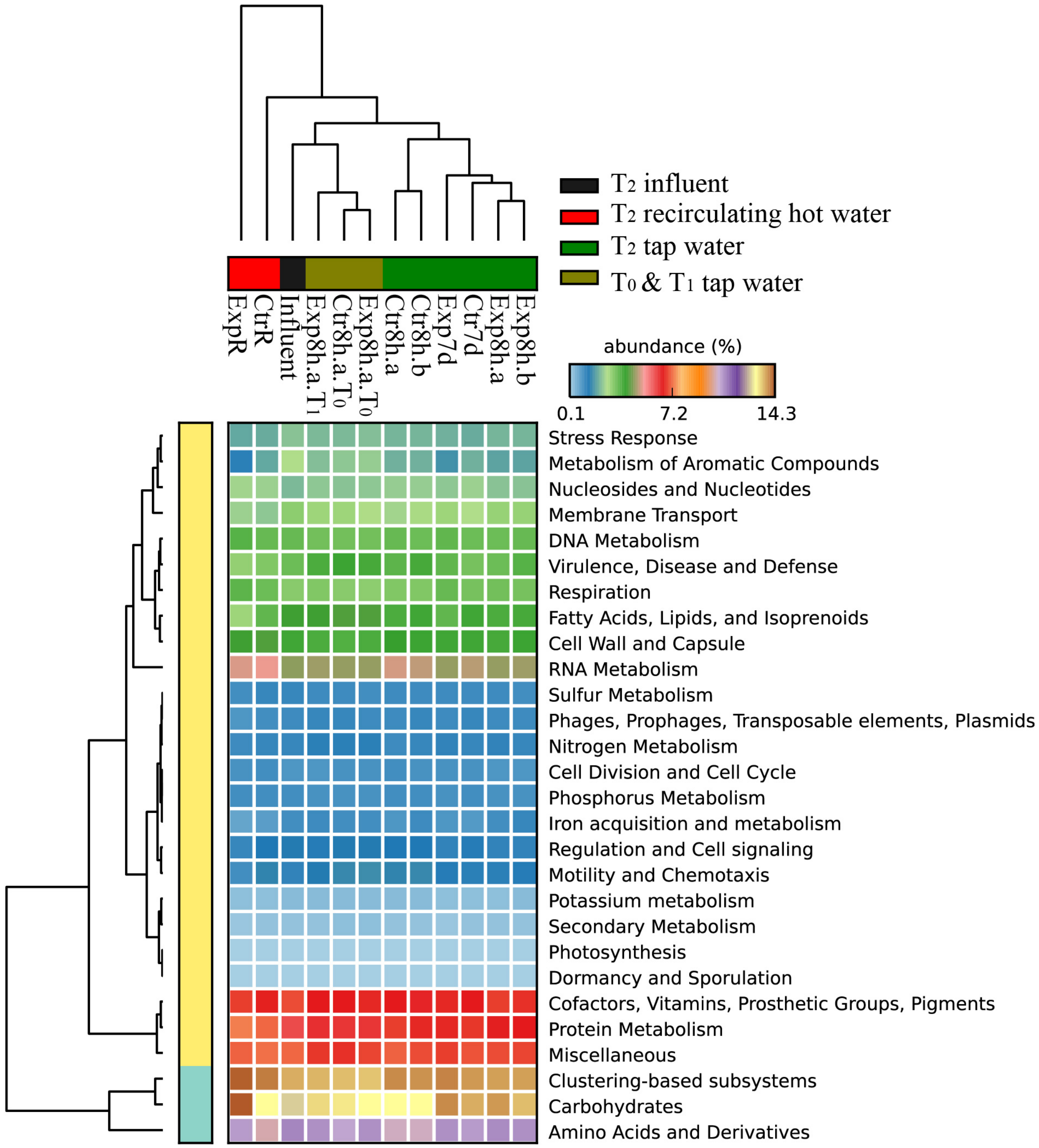

Figure S9. Clustering of all samples based on similarities in proportions of level-1 functions.

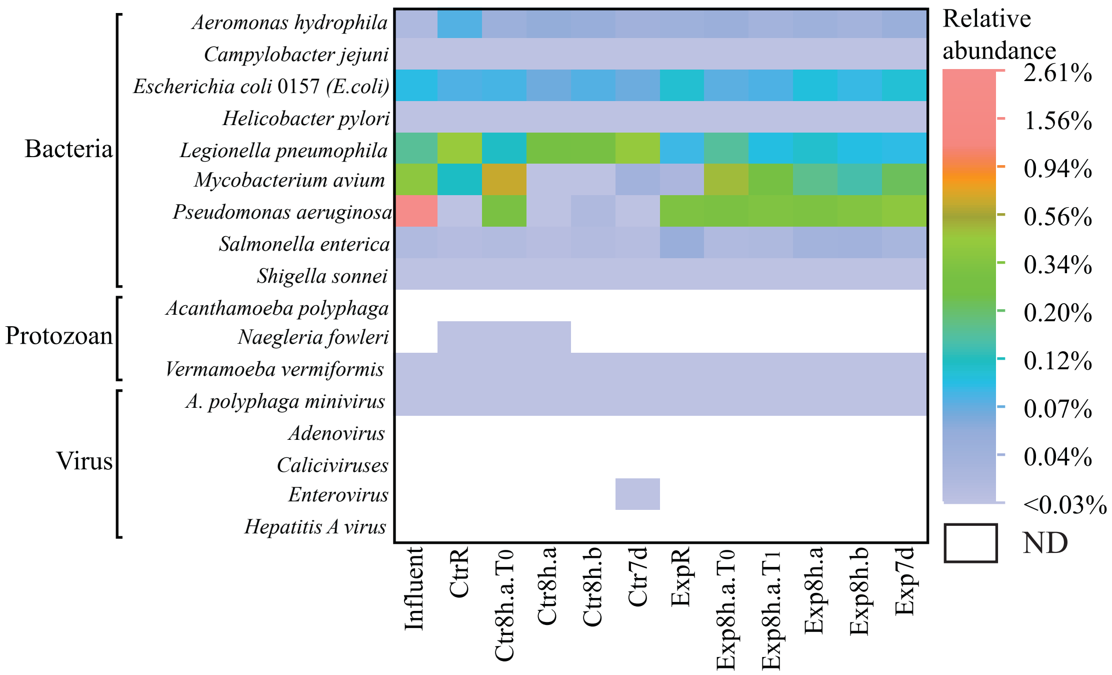

Figure S10. Relative abundances of primary and opportunistic waterborne pathogens listed in Contaminant Candidate List (CCL4) by United States Environmental Protection Agency. Relative abundance of taxa is the count of reads annotated as the taxa normalized by total read counts. ND: not detected.

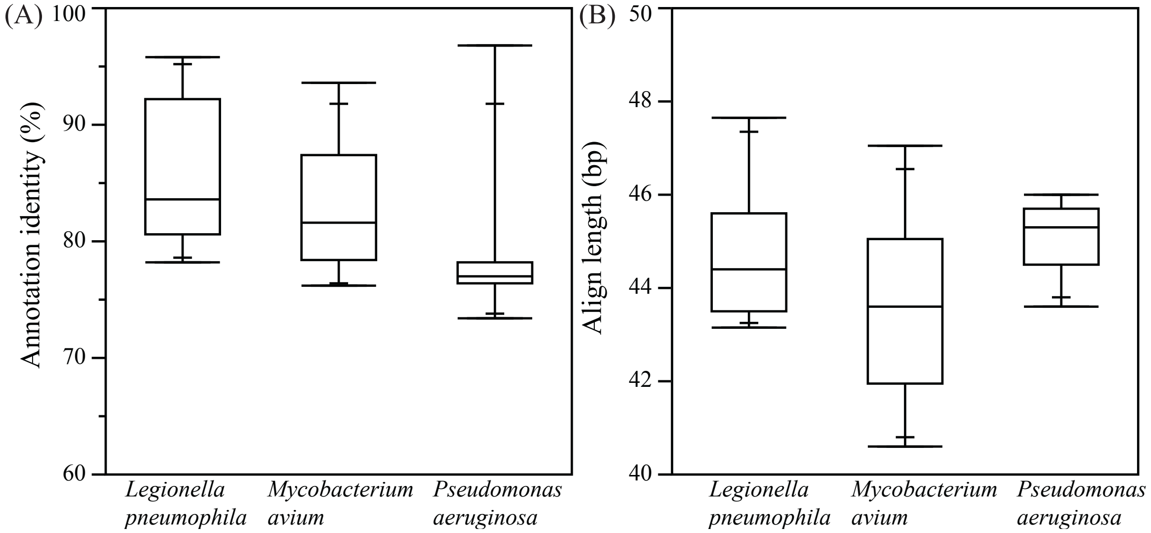

Figure S11. The distribution of annotation identity and align length of reads identified as a particular OP among all samples in this study. Showed in box plots are the maximum, 90% percentile, 75% percentile, median, 25% percentile, 10% percentile, and the minimum values.

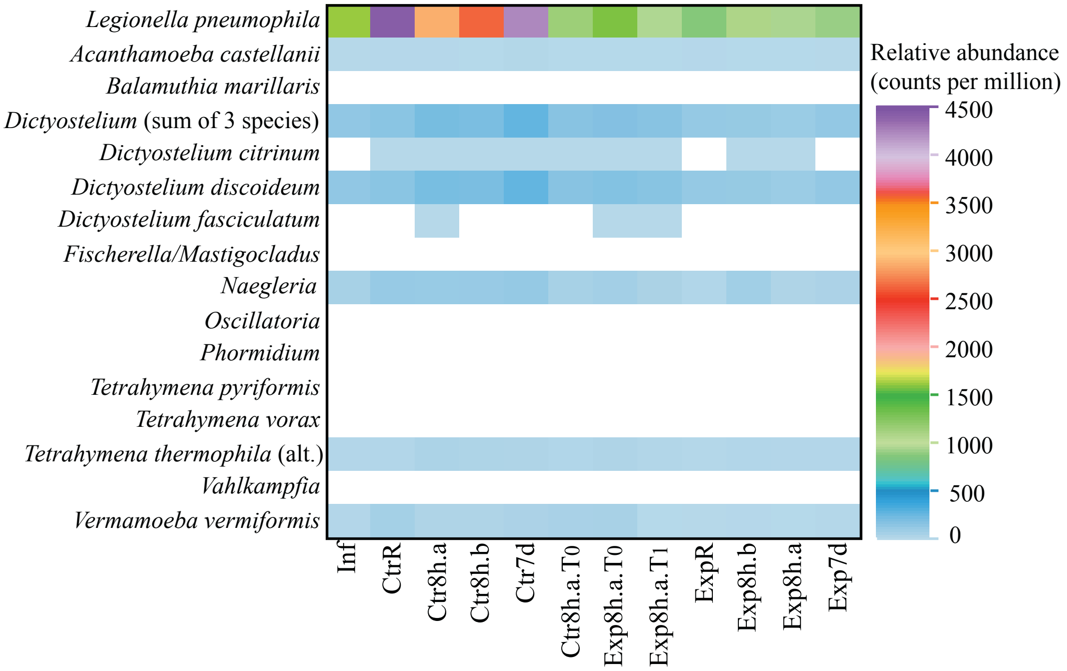

Figure S12. Relative abundances of *L. pneumophila* and protozoa that may be ecological related with *L. pneumophila*. Relative abundance of taxa is the count of reads annotated as the taxa normalized by total read counts. White blocks mean no detection.

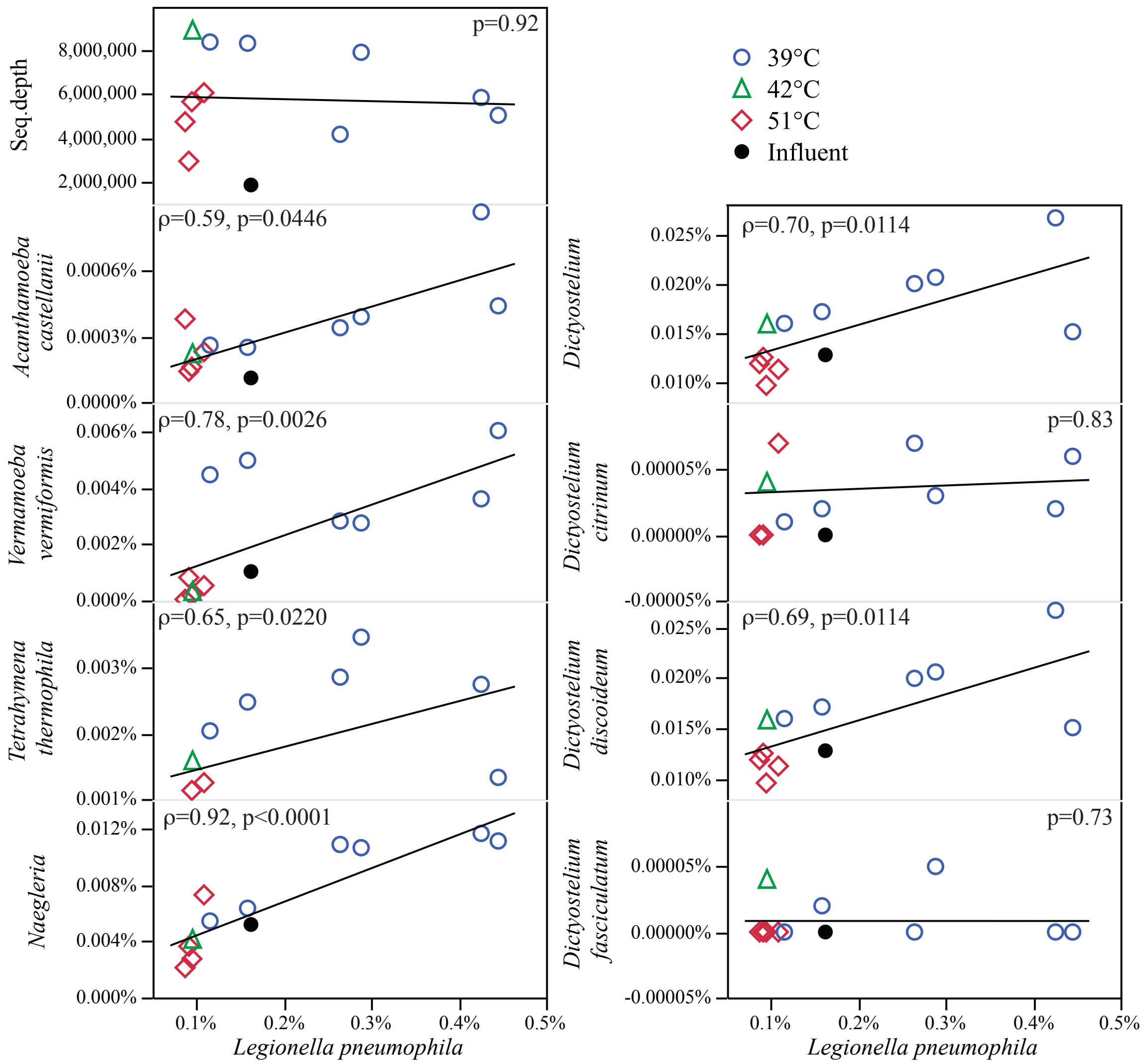

Figure S13. Correlations between the relative abundance of protozoa hosts with *L. pneumophila*. Spearman correlation coefficient ρ and p-values were shown. These correlations were unlikely driven by variable sequence depth, since relative abundance of *L. pneumophila* or protozoan hosts was not correlated with sequence depth (seq. depth). Different temperature settings were illustrated as different symbols.

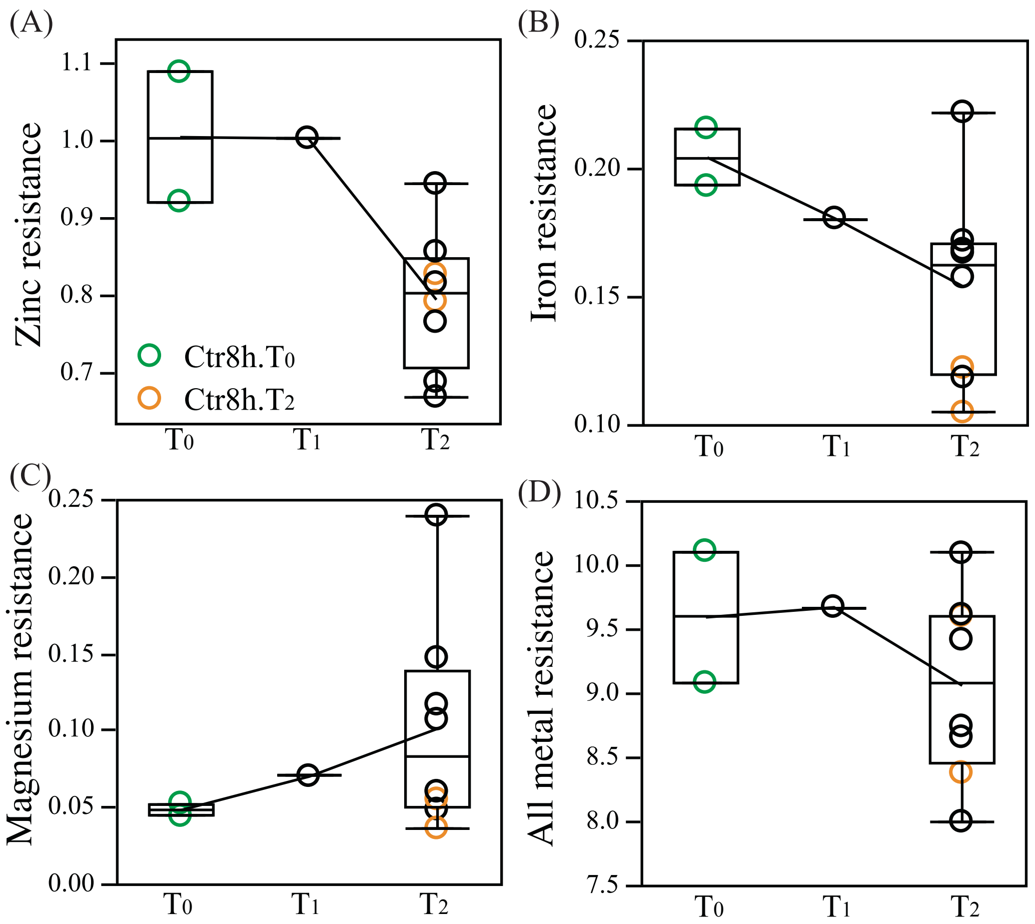

Figure S14. Changes of metal resistance (normalized to the counts of 16S rRNA reads) to **(A)** zinc, **(B)** iron, **(C)** magnesium, and **(D)** all metals with time, from T_0 to T_2. Hot waters from the recirculating line and distal taps of both rigs at the same time-point were grouped together. The same distal taps where hot water stagnated for 8 h from the control rig were color coded as green at T_0 and yellow at T_2 to emphasize soley temporal changes.

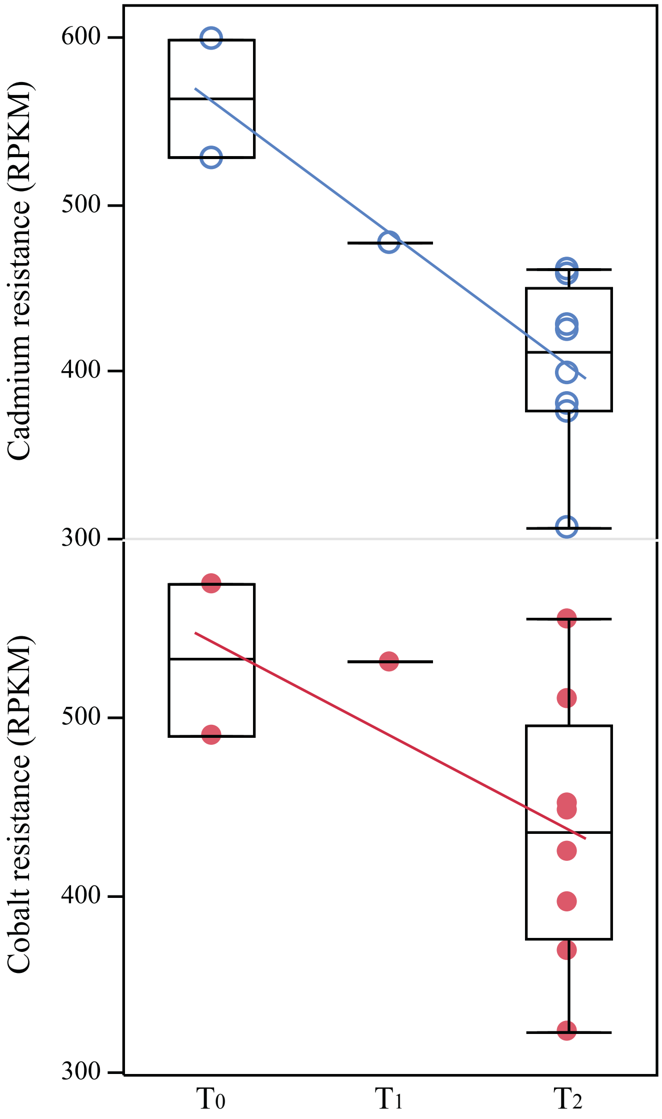

Figure S15. Reduction of reads corresponding to cadmium and cobalt resistance (normalized as reads per kilobase of transcript per million, RPKM) along with time from T_0 to T_2. Hot waters from the recirculating lines and distal taps of both rigs from the same time-point are grouped together.

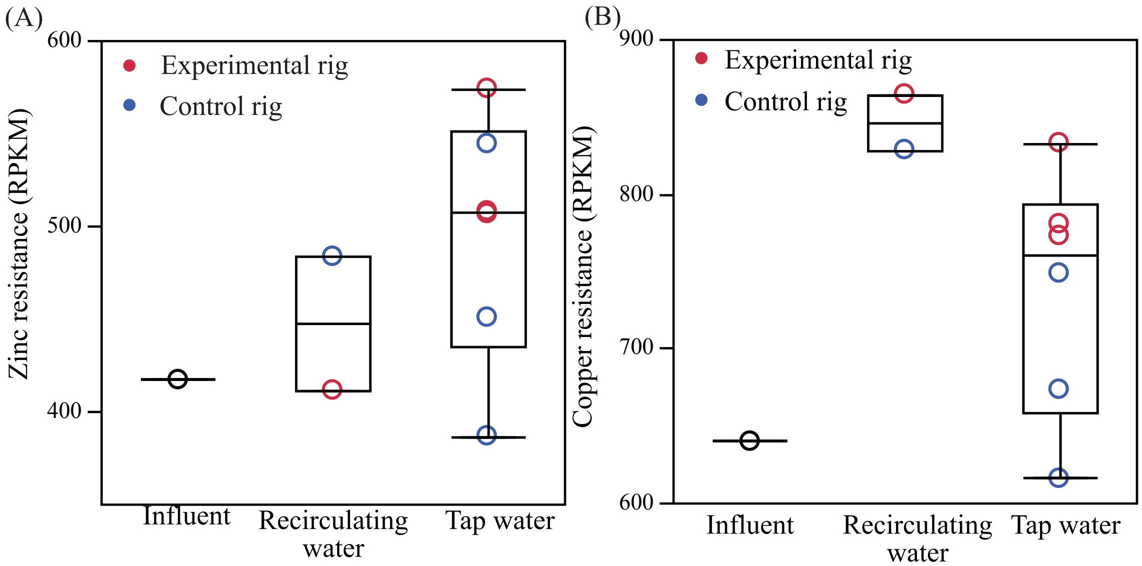

Figure S16. Reads corresponding to zinc and copper resistance, normalized as RPKM (reads per kilobase of transcript per million), increased in recirculating hot water and stagnated distal tap waters in comparison to the influent cold water at T_2 for both experimental rig (red, run at 51°C) and control rig (blue, run at 39°C).

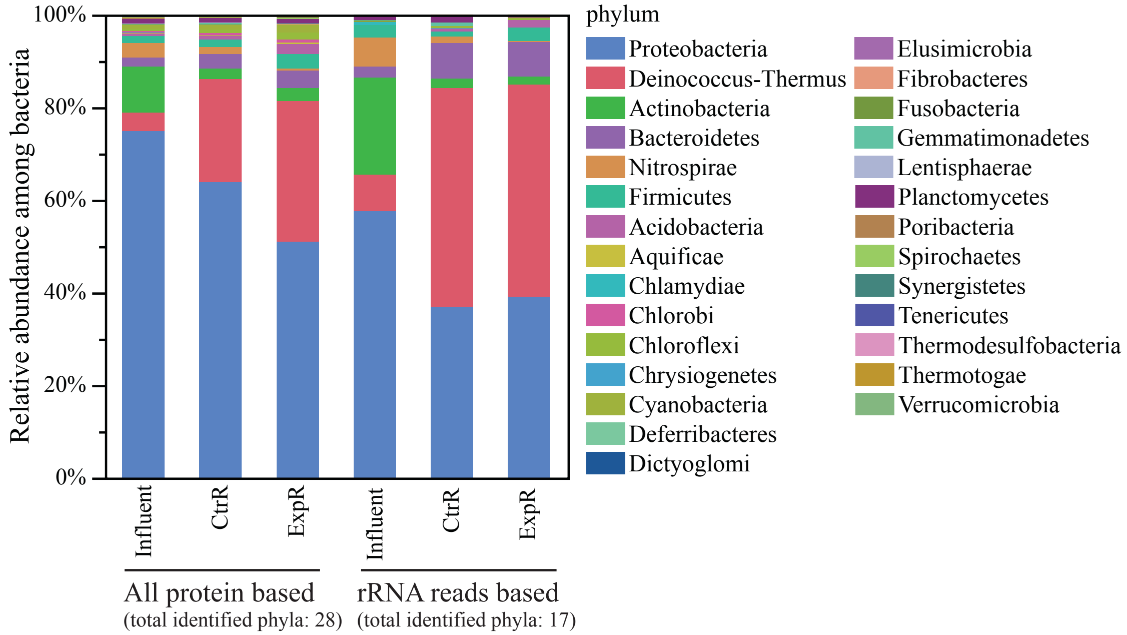

Figure S17. Comparing all protein based with rRNA reads based annotation for phylum composition within Bacteria kingdom. Relative abundances of the phyla were normalized to total number of short reads annotable to bacteria phyla (total number: 1.7-5.6 millions for all protein based method, and 704-4190 for rRNA reads based method.)
